# Plant Wearable Sensors for Data-Driven Precision Agriculture: From Multilevel Sensing to Crop-State Assessment and Adaptive Management

**DOI:** 10.3390/s26196241

**Published:** 2026-10-01

**Authors:** Guangwei Wang, Youchao Gu, Pengfei Zuo, Yue’e Xie, Yuanping Chen

**Affiliations:** 1 School of Physics and Electronic Engineering, Jiangsu University, Zhenjiang 212013, China; 2 Quantum Sensing and Agricultural Intelligence Detection Engineering Center of Jiangsu Province, Zhenjiang 212013, China

**Keywords:** plant wearable sensors, precision agriculture, multimodal sensing, plant phenotyping, crop-state assessment, data-driven decision support

## Abstract

**Highlights:**

**What are the main findings?**

- Physical wearables favor continuous monitoring; biochemical selectivity adds calibration and maintenance demands.
- Crop-state assessment requires explicit physiological targets and growth-stage-specific validation.

**What are the implications of the main findings?**

- Physiological sentinel nodes require replicated sampling and independent tests of spatial representativeness.
- Fusion and assimilation must account for response delays, sensor uncertainty, and leaf-to-canopy scale differences.

**Abstract:**

Plant wearable sensors can resolve physiological responses that soil, weather, and canopy observations capture only indirectly. This critical narrative review evaluates optical, electrochemical, electrical, mechanical, thermal, and acoustic platforms across microenvironmental, growth, biochemical, and stress-related measurements. Crop-state assessment is defined as estimation of management-specific physiological variables, with independent reference measurements and growth-stage-dependent interpretation. The central trade-off is between continuous operation and physiological specificity: physical sensors offer relatively simple monitoring architectures, whereas biochemical sensors provide more selective information but impose greater calibration and maintenance demands. Neither analytical sensitivity nor short-term plant attachment establishes field readiness. We identify requirements for drift control, temporally aligned multimodal fusion, and data assimilation with explicit observation and uncertainty models. Physiological sentinel nodes are proposed as a sampling strategy whose within-plant and field representativeness must be tested before spatial extrapolation. Progress toward adaptive management requires staged validation from organ-level measurements to replicated field trials that demonstrate benefits beyond existing monitoring systems.

## 1. Introduction

Improving agricultural productivity while reducing resource inputs requires increasingly timely and accurate information on crop and field conditions. Accordingly, modern agriculture is shifting from experience-based management toward data-driven and precision-oriented practices, in which sensing technologies provide essential information for irrigation, nutrient management, crop protection, and yield optimization [1]. Soil and meteorological sensors characterize local environmental conditions, while remote sensing provides spatial information on canopy structure, temperature, and spectral responses. Although these approaches are fundamental for identifying spatial variability across agricultural fields, they provide limited access to the dynamic physiological and biochemical responses occurring within individual plants. Consequently, similar environmental or canopy-level observations may correspond to different crop responses, limiting the interpretation of water, nutrient, growth, and health status and the development of timely management decisions [2,3].

Plant wearable sensors provide a plant-centered sensing approach that complements soil, meteorological, and canopy-level observations. Through direct attachment to leaves, stems, and fruits, or minimally invasive integration with internal tissues, these devices extend crop monitoring from external conditions to plant surfaces and endogenous physiological processes [4]. Their close contact with living tissues enables continuous in situ acquisition of microenvironmental, morphological, physiological, and biochemical information while reducing the temporal limitations associated with destructive sampling and laboratory analysis. Advances in flexible electronics, functional materials, micro- and nanofabrication, and biosensing technologies have expanded measurable targets from temperature, humidity, gas concentration, and growth strain to ions, phytohormones, metabolites, volatile organic compounds (VOCs), pathogens, and pesticide-related signals. Collectively, these measurements provide multilevel information on crop water status, nutrient condition, growth dynamics, physiological regulation, and health, forming a plant-centered sensing layer for crop-state assessment in precision agriculture.

In this review, crop-state assessment means estimating a specified physiological condition at a defined organ, plant, or management-zone scale and time horizon. Water status may be evaluated against leaf water potential or stomatal conductance; nutrient status against tissue nutrient concentration or a validated sufficiency range; growth against independently measured organ expansion or biomass; and infection against pathogen-specific diagnostics. Temperature, strain, ion concentration, and hormone trajectories are observations or explanatory variables, not interchangeable measures of these states. Their relationships with the target depend on cultivar, leaf position, developmental stage, and environment. We therefore favor separate, calibrated state estimates over a universal health score. If a composite indicator is needed, its normalization and weights should be specified from the management objective or learned within training data, then tested on independent plants and stages; equal or fixed weights are not assumed.

Despite rapid technological progress, most existing reviews classify plant wearable sensors according to device materials, transduction mechanisms, or individual sensing targets. Such classifications are valuable for understanding sensor design and operation, but provide less insight into how signals acquired at different biological levels can be related to crop physiological status and field management. Plant responses to environmental and management inputs develop through interconnected processes. External conditions first modify the plant microenvironment and subsequently induce changes in surface physical characteristics and growth morphology. At a deeper physiological level, ion transport, hormonal signaling, and metabolic regulation reflect the plant’s internal adjustment to these external changes. Stress-related volatiles, pathogen-associated signals, and pesticide residues provide additional information on crop health, external threats, and crop-protection outcomes. Organizing wearable sensing according to this biological progression can therefore clarify not only what is measured, but also how different signals contribute to crop-state assessment.

For practical application in precision agriculture, however, high sensitivity and flexible attachment alone are insufficient. Sensor outputs must remain reliable under changing field conditions and be linked to physiologically meaningful crop-state variables. Measurements obtained from individual leaves or plants are also influenced by developmental stage, canopy position, local microclimate, genotype, and spatial heterogeneity across the field. Their broader management value therefore depends on long-term operational stability, physiological calibration, representative sensor placement, wireless data transmission, and integration with soil, meteorological, remote-sensing, and other field information. These requirements are critical for translating plant-level measurements into reliable crop-state assessment and ultimately into field-scale management.

Accordingly, this review is organized around the data pathway linking plant-attached measurement to agricultural action. Optical, electrochemical, electrical, mechanical, acoustic, and thermal platforms are first examined as a plant-centered data-acquisition layer. Their applications are then evaluated as sources of time-resolved microenvironmental, phenotypic, physiological, biochemical, and threat-related information. The analysis subsequently considers how multimodal wearable observations can be quality-controlled, fused with soil, meteorological, unmanned aerial vehicle (UAV) or robotic, remote-sensing, and crop-model data, and translated into crop-state estimates. Finally, the review discusses representative field deployment, data assimilation, digital twins, and adaptive irrigation, fertilization, and crop-protection workflows. By moving from sensing mechanisms to data integration and management feedback, this framework clarifies the specific contribution of plant wearables to data-driven precision agriculture.

### Review Scope, Literature Selection, and Analytical Framework

This review examines how plant wearable sensors translate physical and biochemical signals into information for crop-state assessment and precision management. It focuses primarily on developments published from 2016 to 2026, while earlier studies are retained where they provide foundational sensing principles or essential methodological context. The discussion connects sensing mechanisms and sensor–plant interfaces with physiological interpretation, data integration, and agricultural applications. Comparative analysis considers measurement performance, monitoring duration, environmental interference, drift, plant disturbance, and validation under laboratory and field conditions. Particular attention is given to whether the available evidence demonstrates analytical feasibility, sustained on-plant monitoring, or measurable benefits for crop management.

A supplementary literature search was conducted in the Web of Science Core Collection using the Topic field and a publication-year range of 2016–2026. Plant- and crop-related terms were combined with device terms, including wearable, plant-attached, on-leaf, in planta, implantable, microneedle, and flexible, together with sensor, sensing, or monitoring. In addition, bibliographic details of the cited references were cross-checked using digital object identifiers (DOIs), publisher, and available indexing information, with X-MOL used as a supplementary literature-discovery platform where applicable. This supplementary search and reference check were used to verify the coverage of the existing thematic bibliography and identify potential omissions; they do not retrospectively define a systematic-review dataset or a single final set of eligible studies.

For the qualitative synthesis, original experiments conducted on living plants provide the primary evidence for evaluating sensor performance under the reported conditions, whereas studies on materials, analytical methods, plant physiology, remote sensing, data processing, and modelling provide methodological or contextual support. The review therefore evaluates representative evidence across the sensing-to-management pathway rather than claiming exhaustive coverage of all related literature.

**Figure 1:**
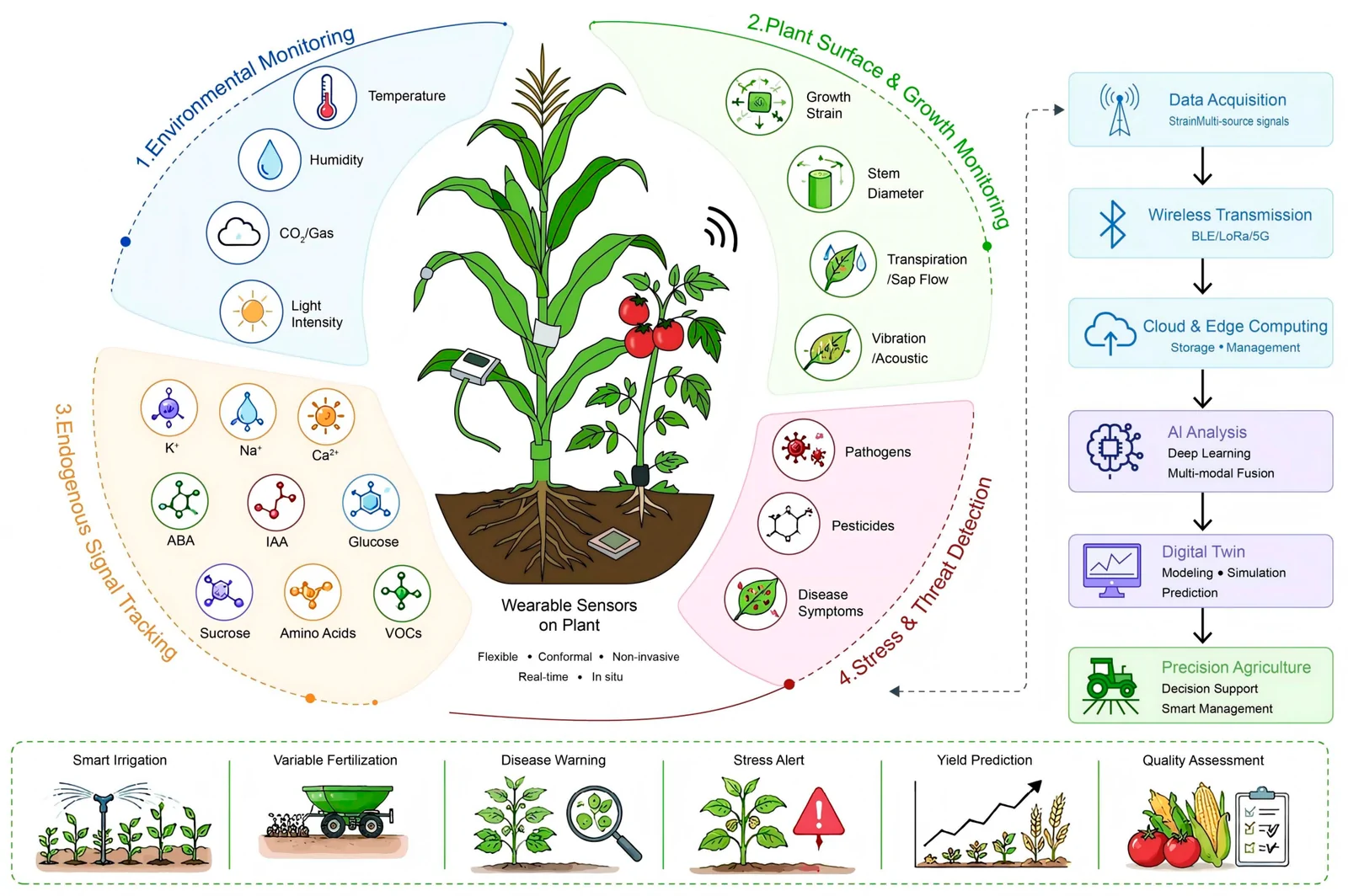
Overview of Plant Wearable Sensor Classification and Information Flow. The diagram links environmental, growth, chemical, and stress observations to data acquisition, interpretation, and management. Arrows indicate conceptual information flow rather than experimentally validated causal relationships.

Figure 1 connects the major plant wearable sensing technologies with the progression of crop information from environmental exposure and surface responses to internal physiological and chemical regulation, and further illustrates how these multilevel signals can be integrated for crop-state assessment and precision management under field conditions.

## 2. Plant Wearable Sensing Technologies for Precision Agriculture

The core objective of precision agriculture is the timely and in situ characterization of crop growth status and stress responses. Achieving this goal increasingly relies on sensing technologies capable of converting environmental conditions, plant phenotypes, and endogenous physiological signals into measurable outputs. For this review, plant wearable sensors are grouped into optical, electrochemical, electrical, mechanical, acoustic, and thermal systems [5]. These groups combine readout mechanisms and measured responses and are not mutually exclusive: mechanical and thermal changes may be read electrically or optically. The grouping supports comparison of crop information, physiological specificity, and deployment requirements rather than a strict taxonomy of transduction mechanisms.

### 2.1. Optical Wearable Sensors

Optical wearable sensors exploit photoelectric effects or changes in optical properties arising from light–matter interactions to convert plant-related responses into measurable signals. Their transduction mechanisms include photoconductive, photogating, and photovoltaic effects, as well as changes in absorption, scattering, fluorescence, or optical transmission associated with plant composition and physiological status [6,7].

Conventional optical technologies, including near-infrared (NIR), Raman, and fluorescence spectroscopy, can provide rapid and sensitive detection, often without destructive sampling, and have been widely applied to crop nutrition, quality assessment, pesticide residues, diseases, and abiotic stress, as summarized in Table 1 [8]. When integrated with unmanned aerial vehicles or ground-based remote-sensing platforms, however, these approaches primarily characterize canopy- or organ-scale optical responses and may not fully resolve physiological differences among individual plants or leaves. Direct integration of optical sensors with plant organs therefore provides a complementary strategy for acquiring continuous information closer to the source of plant physiological responses [9,10]. Current plant-wearable optical technologies mainly include colorimetric, surface-enhanced Raman scattering (SERS), fluorescence, and optical-fiber sensors [11,12,13,14,15].

**Figure 2:**
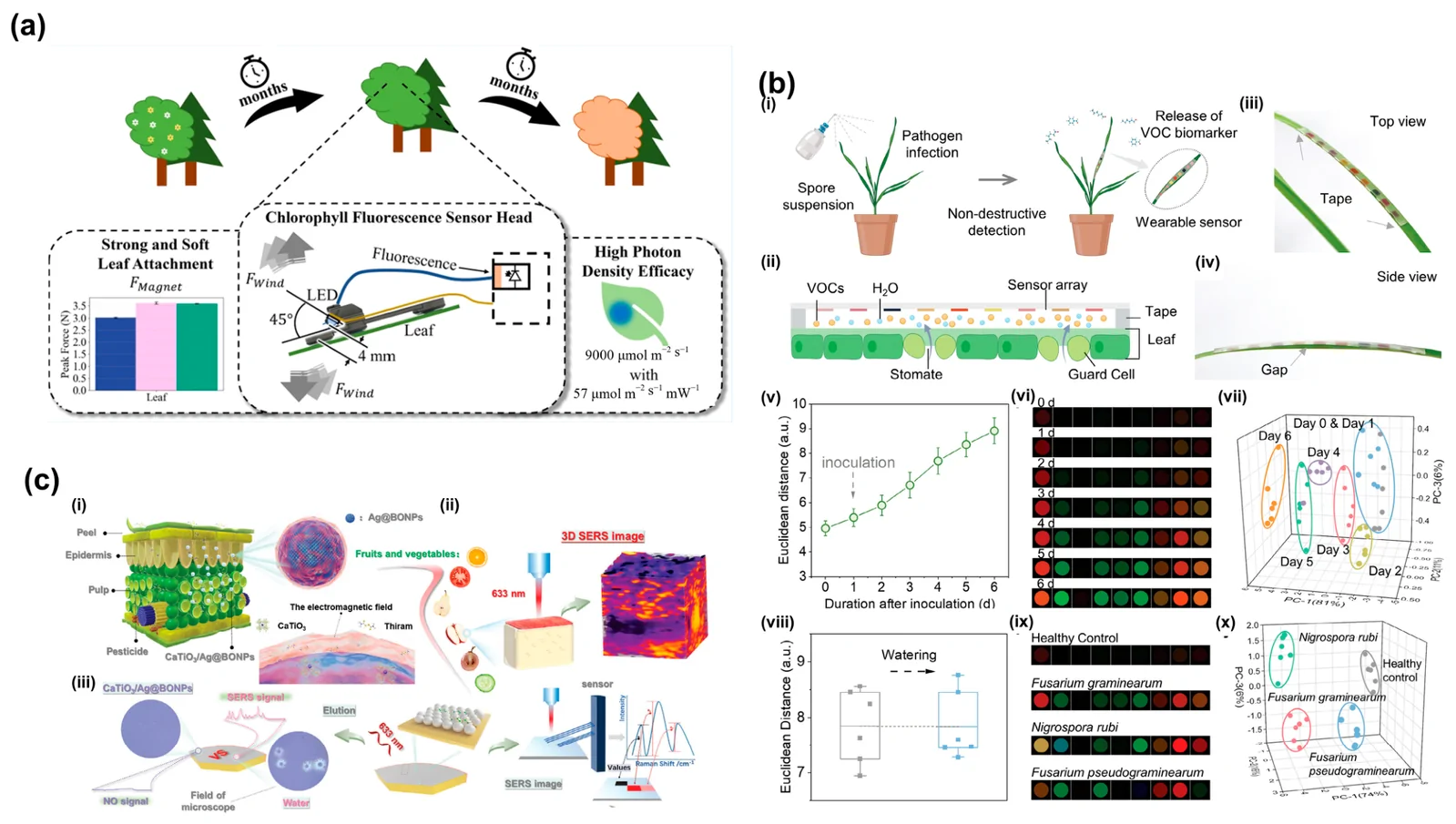
Representative optical sensing technologies for plant monitoring. (**a**) On-leaf chlorophyll fluorescence sensor [27]. (**b**) Wearable volatile organic compound (VOC) sensor based on surface-functionalized metal–organic framework (MOF) inks [28]. (**c**) Surface-enhanced Raman scattering (SERS) imaging for pesticide detection in crop tissues [29]. In (**b**), (**i**) shows inoculation and sensing, (**ii**) the sensor–leaf interface, (**iii**,**iv**) top and side views, (**v**) the response over time, (**vi**) color-difference profiles, (**vii**) principal-component analysis over time, (**viii**) the watering comparison, and (**ix**,**x**) color-difference profiles and principal-component analysis for different pathogen treatments. In (**c**), (**i**–**iii**) show pesticide penetration, three-dimensional SERS imaging, and pesticide adsorption/removal and imaging. Arrows indicate the illustrated sensing or processing sequence; in (**a**), they also indicate seasonal progression, wind loading, and the optical path. Colored spots in (**b**) represent differential sensor responses, and plot colors distinguish the labeled days or pathogen groups; false colors in (**c**) represent spatial SERS responses. Panels (**a**–**c**) are adapted from the respective sources cited above under the terms of the Creative Commons Attribution 4.0 International License (CC BY 4.0).

Colorimetric, SERS, and fluorescence wearable sensors integrate flexible substrates with chromogenic reagents, noble-metal nanostructures, or fluorescent probes to convert chemical and biochemical changes at plant surfaces into colorimetric, Raman, or fluorescence signals, respectively (Figure 2a–c). These optical responses enable in situ detection of plant exudates, metabolites, nutrient-related indicators, stress-associated compounds, pesticide residues, and disease-related signals [16,17]. Their flexible configurations, rapid responses, high sensitivity, and compatibility with visual or portable signal acquisition facilitate direct plant-level monitoring and rapid field assessment [18,19,20].

By contrast, optical-fiber sensors are particularly suitable for continuous monitoring of plant growth and local microenvironmental dynamics. Fiber Bragg grating (FBG) sensors detect stem or petiole deformation, whereas micro- and nanofibers coated with humidity- or gas-sensitive materials can monitor boundary-layer humidity and gaseous compounds near leaf surfaces. Multiple sensing units can be integrated along a single optical fiber, enabling measurements at different positions on the same plant and supporting continuous monitoring of dehydration and diurnal growth dynamics. Passive interrogation and corrosion-resistant materials are useful for extended monitoring, but field suitability still depends on fiber routing, temperature compensation, interrogation hardware, and measured effects on plant function [21,22].

Overall, optical wearable sensors combine flexible and lightweight configurations with rapid, nondestructive, and in situ detection, enabling direct acquisition of chemical, physiological, growth, and microenvironmental information from living plants. In precision agriculture, these plant-level measurements can complement unmanned aerial vehicles, ground-based remote sensing, soil and meteorological monitoring, and wireless data transmission. Remote sensing provides broader spatial information across agricultural fields, whereas wearable optical sensors supply continuous observations from selected plants, helping relate field-scale variability to corresponding physiological or environmental responses. This complementary framework can support nutrient diagnosis, irrigation management, stress and disease warning, targeted crop protection, and pesticide-residue assessment [23,24,25,26].

**Table 1:** Spectroscopic Techniques for Agricultural Sensing.

Technique	Principle	Target	Advantages	Limitations	Reference
NIR	Vibrational absorption	Leaf water & biochemical status	Rapid analysis; multi-parameter detection	Model-dependent	[30]
LIBS	Laser ablation	Plant tissue elemental nutrition	Multi-element analysis; no sample preparation	Matrix-dependent	[31]
Raman	Inelastic scattering	Leaf molecular metabolites & stress markers	Non-destructive; no sample preparation	Fluorescence interference	[19]
Fluorescence	Photon excitation	Photosynthetic activity & plant stress state	High sensitivity; in vivo applicability	Environmental interference	[32]

Abbreviations: near-infrared spectroscopy (NIR); laser-induced breakdown spectroscopy (LIBS).

### 2.2. Electrochemical Wearable Sensors

**Figure 3:**
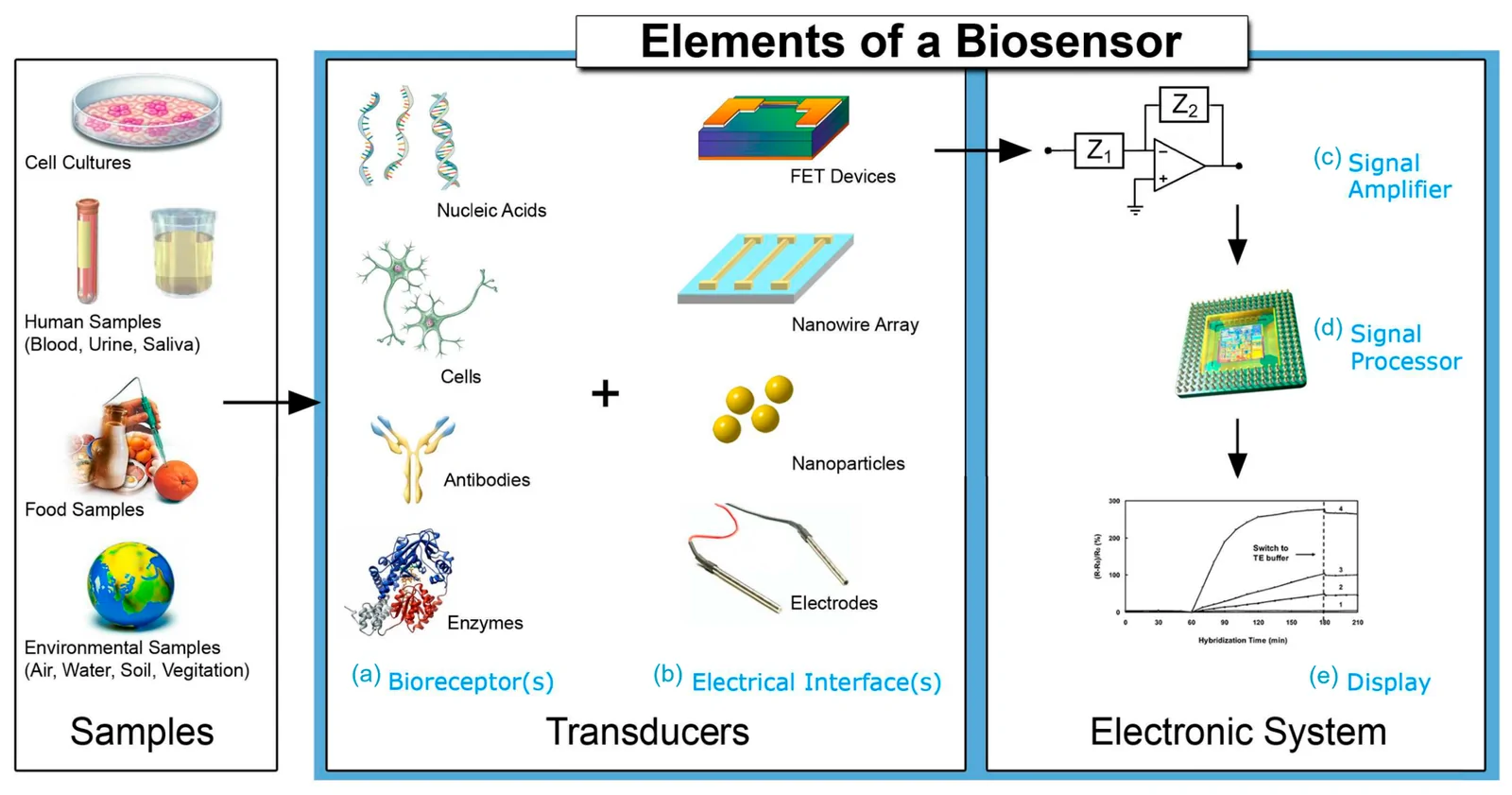
Elements and selected components of a typical biosensor [48]. The diagram includes a field-effect transistor (FET) readout. Labels (**a**–**e**) identify bioreceptors, electrical interfaces, signal amplification, signal processing, and display, respectively. In the illustrative display, curves 1–4 correspond to 1.0 nM non-complementary DNA (control) and 25 fM, 100 fM, and 1.0 nM target DNA, respectively, as described in Ref. [48]. Z1 and Z2 denote amplifier impedances. Adapted from the source cited above under the terms of the Creative Commons Attribution License (CC BY). The diagram illustrates a general recognition–transduction–readout chain; the sample examples are not all plant-wearable applications.

Electrochemical wearable sensors provide direct access to chemical information at plant surfaces and within living tissues. They convert analyte-dependent reactions or binding events into changes in current, potential, or impedance, allowing plant biochemical signals to be recorded using compact electrical readout systems [33]. By integrating flexible electrodes and substrates, these sensors can be attached to leaves, stems, or fruits or coupled with minimally invasive sampling structures. Unlike soil and meteorological sensors, which characterize external growth conditions, electrochemical wearables measure chemical changes associated with the plant response itself, including ion uptake, metabolic regulation, stress signaling, pathogen infection, and pesticide exposure. Accordingly, electrochemical wearables can determine whether spatial variability identified by field-scale monitoring is accompanied by corresponding physiological or biochemical changes in individual plants (Figure 3).

The measurable target and signal specificity of wearable electrochemical sensors are determined primarily by the recognition interface. These sensors can be broadly divided into direct sensing systems and platforms incorporating target-specific recognition elements [34]. Table 2 summarizes five representative strategies—direct, enzymatic, immunoelectrochemical, aptamer-based, and molecularly imprinted sensing—together with their recognition principles, principal targets, advantages, and limitations. Direct electrochemical sensors monitor ions, reactive oxygen species, and selected small-molecule metabolites through analyte-induced changes at the electrode interface. Their simple configuration facilitates miniaturization and continuous monitoring, although limited molecular selectivity can complicate signal interpretation in complex plant fluids [35,36].

For targets requiring greater molecular specificity, enzymes, antibodies, or aptamers can be immobilized on the electrode surface to selectively recognize metabolites, phytohormones, and pathogen-related compounds [37,38,39]. Molecularly imprinted polymers (MIPs), functionalized nanomaterials, and other synthetic recognition interfaces provide more chemically robust alternatives for repeated in situ measurements, although their selectivity and stability must still be validated in actual plant matrices. These recognition strategies extend wearable electrochemical sensing to pesticide residues, heavy-metal ions, plant VOCs, and other biologically or agriculturally relevant compounds, thereby broadening access to crop exposure, endogenous regulation, infection, and crop-protection outcomes [40,41,42].

The development of flexible electrode materials has further enabled these recognition strategies to operate on living plants [43]. Conventional metal, carbon-paste, and conductive-polymer electrodes have increasingly been supplemented by graphene, carbon nanotubes (CNTs), MXenes, conductive hydrogels, and metal nanoparticles, which combine electrical conductivity with the mechanical conformity required for attachment to irregular and growing plant organs. Hybrid sensing layers can maintain electrical contact during leaf movement, stem expansion, and field-induced deformation, allowing analyte-dependent changes to be followed as continuous time-series signals rather than as isolated measurements [44]. For precision agriculture, this continuity is important because the timing, persistence, and recovery of a plant response can be more informative for management than a single concentration measurement.

Wearable electrochemical measurements can further be interpreted together with georeferenced remote-sensing observations and local soil and meteorological data. While remote and proximal sensing characterize the spatial extent and possible environmental causes of field variability, electrochemical wearables provide direct evidence of plant-level changes in nutrient- or water-related ions, stress signaling, pathogen-associated targets, and pesticide residues. These observations may support targeted management when the measured analyte has a validated relationship with the crop state and intervention of interest [45,46,47].

**Table 2:** Electrochemical Recognition Mechanisms for Wearable Sensors.

Technique	Principle	Target	Advantages	Limitations	Reference
Direct Electrochemical	Bare electrode	Leaf ions	Fast; simple	Limited selectivity	[49]
Enzymatic Electrochemical	Enzyme reaction	Metabolites	High sensitivity	Enzyme deactivation	[50]
Immunoelectrochemical	Antibody binding	Pathogens	High specificity	High cost; poor stability	[51]
Aptamer-Based Electrochemical	Aptamer binding	Hormones	programmability	Complex fabrication	[52]
Molecularly Imprinted Electrochemical	Imprinted sites	Pesticides	Stable; cheap	Less selective	[53]

### 2.3. Electrical Wearable Sensors

Electrical wearable sensors convert changes in conductivity or dielectric properties into directly readable resistance or capacitance signals. Their simple structures and compatibility with flexible substrates facilitate attachment to leaves, stems, and fruits, although physiological disturbance requires independent evaluation. Unlike electrochemical sensors, they generally do not rely on target-specific recognition elements, but instead track deformation, water-related changes, and variations at the plant–sensor interface associated with growth and transpiration. These characteristics make them suitable for continuous plant-level monitoring within precision-agriculture systems.

**Figure 4:**
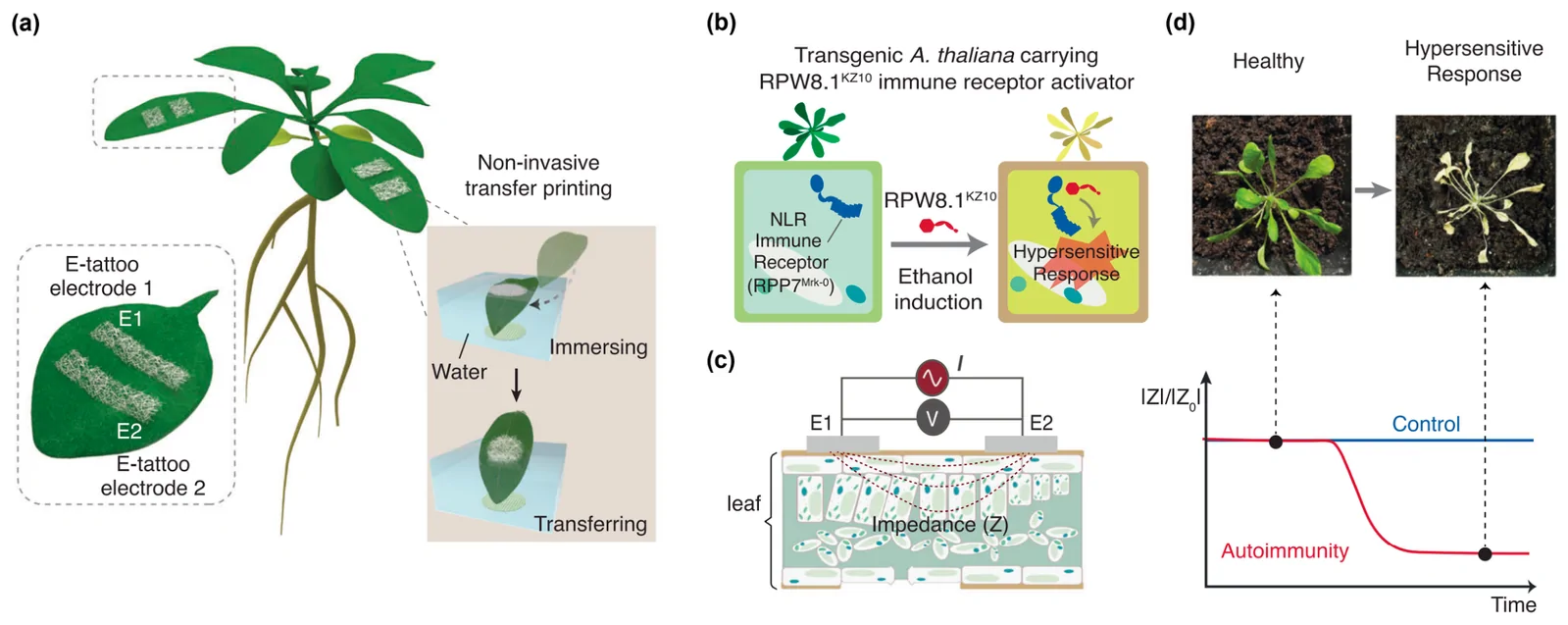
Schematic illustration of an electronic-tattoo sensor for plant impedance monitoring [55]. (**a**) Transfer printing of electronic-tattoo electrodes onto leaves. (**b**) Induction of a hypersensitive immune response in transgenic Arabidopsis thaliana. (**c**) Leaf impedance measurement between electrodes E1 and E2. (**d**) Schematic comparison of normalized impedance in control and autoimmune plants. Arrows indicate transfer steps, immune-response induction, or the correspondence between plant condition and impedance response; blue and red traces denote control and autoimmunity, respectively. Adapted from the source cited above under the terms of the Creative Commons Attribution 4.0 International License (CC BY 4.0).

Resistive wearable sensors respond to changes in conductive pathways caused by tissue expansion, bending, leaf movement, or variations at the plant–sensor interface (Figure 4). Metallic conductors and strain gauges used in early devices have been supplemented by graphene, CNTs, MXenes, conductive polymers, and metallic nanowires, while porous structures and serpentine interconnects improve conformity during plant deformation. Rather than merely measuring strain, these devices provide continuous records of stem or fruit growth, leaf movement, and transpiration-related deformation. Such time-series signals can reveal sustained changes in crop growth and water-related responses that may not be captured by periodic phenotyping alone. Field interpretation nevertheless requires stable attachment and compensation for environmental effects on resistance.

**Figure 5:**
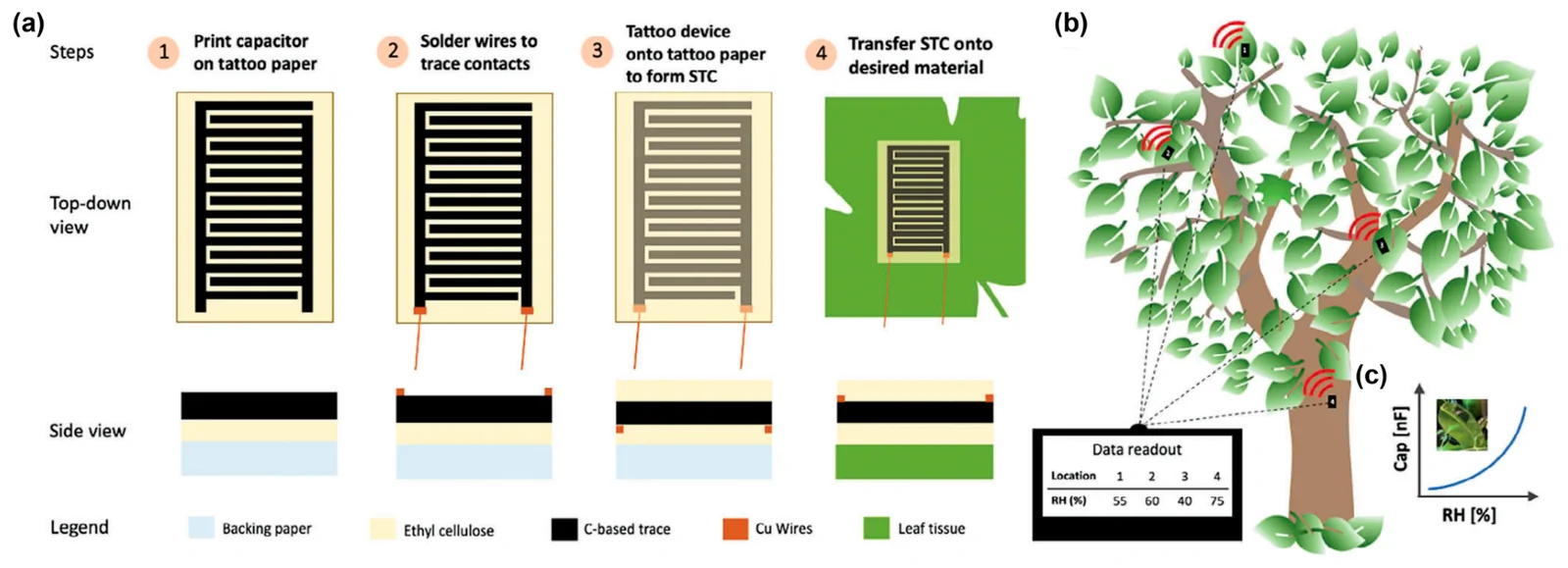
Schematic illustration of a capacitive sensor for relative-humidity monitoring at the leaf surface [56]. (**a**) Fabrication and transfer of a sandwich tattoo capacitor (STC): steps 1–4 comprise capacitor printing, wire connection, assembly on tattoo paper, and transfer to the target surface. (**b**) Schematic deployment at four canopy positions with corresponding relative humidity (RH) readouts. (**c**) Illustrative capacitance response to RH. Layer colors in (**a**) are identified in the embedded legend; Cap denotes capacitance. Adapted from the source cited above under the terms of the Creative Commons Attribution 4.0 International License (CC BY 4.0).

Capacitive wearable sensors detect changes in plant water content, transpiration, or ambient humidity through variations in dielectric properties (Figure 5). Parallel-plate, interdigitated, and flexible microstructured configurations are commonly fabricated using dielectric layers such as polydimethylsiloxane (PDMS), polyimide (PI), Ecoflex, or hydrogels together with flexible electrodes based on silver nanowires, liquid metals, or conductive composites. These structures can be adapted to leaf surfaces for monitoring water status, leaf wetness, transpiration dynamics, fruit ripening, and local humidity, while hydrogels further improve interfacial compatibility and moisture response [54].

In field applications, electrical wearable measurements can be interpreted together with soil-moisture, meteorological, and remote-sensing data to distinguish external water availability from the crop response itself. Resistive and capacitive sensors provide complementary information on crop deformation, leaf wetness, transpiration, and water-related dynamics, helping relate plant-level responses to broader field variability. These measurements can support irrigation assessment and post-treatment evaluation, although their practical value depends on stable sensor–plant contact and appropriate environmental compensation.

### 2.4. Other Wearable Sensing Technologies

Beyond spectral, chemical, and electrical signals, plants also generate deformation, vibration, and temperature responses associated with growth, water transport, and stress. Mechanical, acoustic, and thermal wearable sensors capture these physical signals directly from living organs, providing continuous plant-level measurements that complement field-scale observations from remote sensing and environmental information from soil and meteorological sensors.

**Figure 6:**
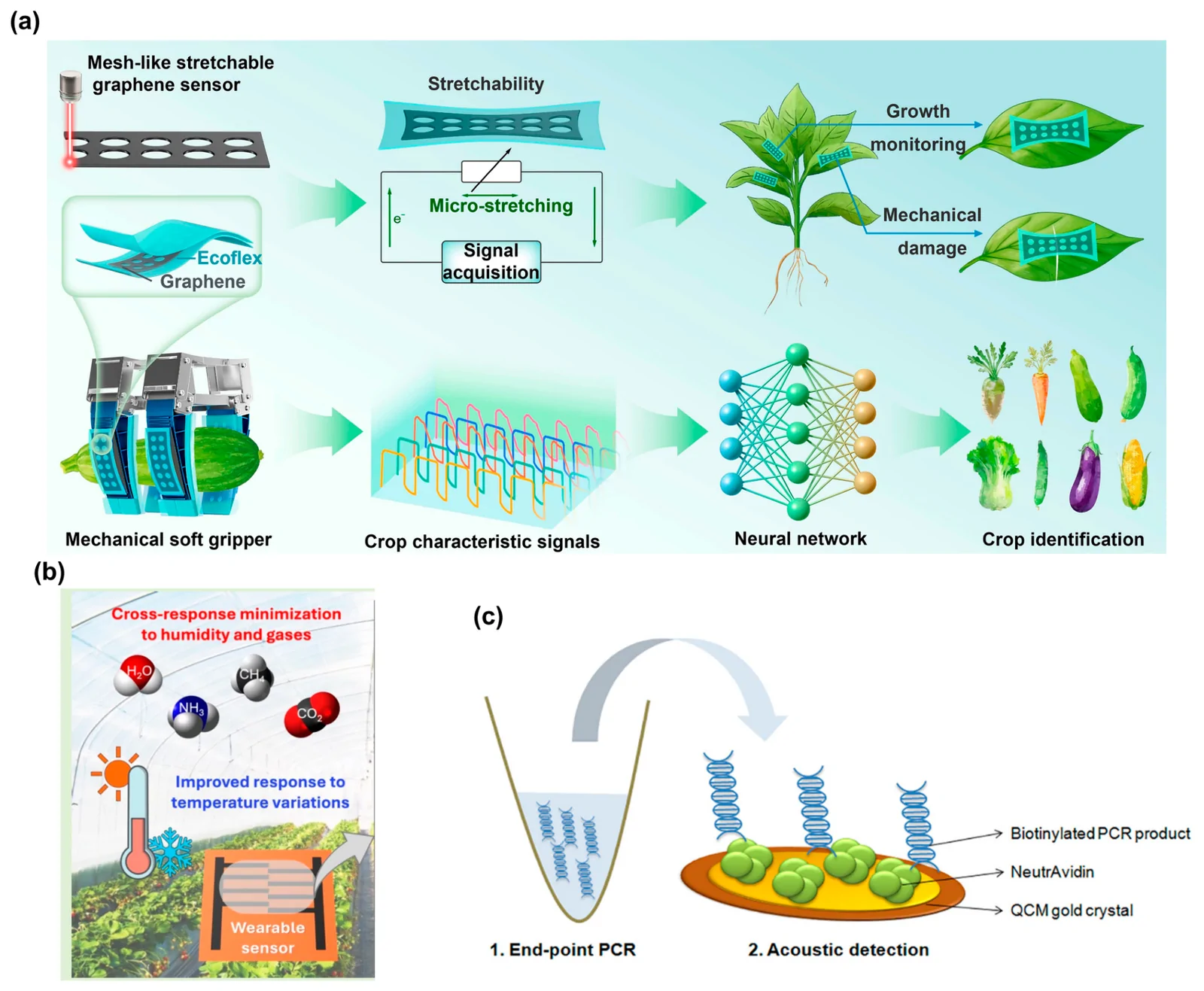
Mechanical, thermal, and acoustic sensing approaches for plant monitoring. (**a**) Stretchable graphene strain sensor for plant growth and mechanical-damage monitoring and crop recognition [59]. (**b**) Temperature sensor based on poly(3,4-ethylenedioxythiophene):polystyrene sulfonate (PEDOT:PSS) and a copper metal–organic framework (Cu-MOF) [60]. (**c**) Quartz crystal microbalance (QCM) detection of bacterial pathogen DNA amplified by polymerase chain reaction (PCR) [61]. In (**a**), arrows connect sensor fabrication, deformation-induced electrical readout, plant monitoring, and crop identification; circuit arrows denote electron flow. In (**b**), the upward arrow denotes an enhanced temperature response. In (**c**), the curved arrow indicates transfer of amplified products to the QCM surface, and leader arrows identify the surface components. Panels (**a**–**c**) are adapted from the respective sources cited above under the terms of the Creative Commons Attribution 4.0 International License (CC BY 4.0). Panel (**c**) uses amplified samples rather than continuous in planta acoustic recording.

Mechanical wearable sensors detect strain, pressure, and deformation generated by stem thickening, leaf expansion, fruit growth, or external stimulation (Figure 6a). Depending on the transduction layer, these changes are converted into resistance, capacitance, or voltage signals, allowing crop growth trajectories and short-term contraction–expansion patterns to be monitored continuously [57]. These continuous deformation signals can reveal persistent growth inhibition and water-related changes that may be missed by periodic phenotyping, providing a direct plant-level indicator of crop growth dynamics and recovery.

Acoustic biosensing also includes quartz crystal microbalance assays for amplified pathogen deoxyribonucleic acid (DNA) (Figure 6c). This sample-based diagnostic approach differs from listening to plant acoustic emissions and does not demonstrate a wearable cavitation sensor. For emission monitoring, wind and mechanical disturbances require rejection before signals can be interpreted alongside soil moisture and weather. Thermal wearable sensors attach flexible thermistors or temperature-sensitive materials to leaves or stems to record local temperature and its temporal variation (Figure 6b). Leaf temperature reflects the interaction between environmental heating and transpirational cooling, while temperature differences along stems can also indicate sap-flow dynamics. When interpreted together with canopy-temperature patterns and local soil-water measurements, these signals can help distinguish environmental heating from plant-specific reductions in transpiration and provide additional information for irrigation assessment and post-irrigation evaluation [58].

Together, mechanical, acoustic, and thermal sensing modalities add direct information on physical plant responses that is not captured by chemical or spectral measurements alone. By continuously capturing deformation, acoustic emissions, and local temperature dynamics, these sensors can reveal changes in crop growth, water transport, and stress responses that are not fully resolved by periodic or field-scale observations. Their integration with environmental and remote-sensing information can improve crop-state interpretation, although practical application still depends on stable plant–sensor contact, representative placement, and calibration against reference physiological measurements.

Together, optical, electrochemical, electrical, mechanical, acoustic, and thermal wearable sensors provide complementary access to the spectral, biochemical, electrical, and physical responses of crops, as summarized in Table 3. These categories overlap: a mechanical strain sensor may use electrical or optical transduction, and a gas sensor may use an electrochemical or chemiresistive interface. Comparisons should therefore distinguish the physiological target from the readout mechanism [62,63].

**Table 3:** Comparison of sensing modalities for crop-state assessment and deployment.

Modality	Crop Information	Strength	Field Constraint	Suitable Role	Reference
Optical	Spectral traits; fiber strain	Rich readout; passive fibers	Illumination, fouling, interrogation	Trait assessment; fiber monitoring	[64]
Electrochemical	Ions and biochemical analytes	Analyte-selective interfaces	Reference drift, fouling, matrix effects	Targeted chemical measurement	[34]
Electrical	Hydration and interface responses	Simple, frequent readout	Cross-sensitivity; contact drift	Continuous response tracking	[65]
Mechanical	Expansion, contraction, movement	Direct deformation measurement	Growth, hydration, attachment overlap	Growth and recovery assessment	[66]
Acoustic	Emission events; QCM DNA assays	Event or molecular readout	Noise for emissions; preparation for assays	Screening; sample-based diagnosis	[61]
Thermal	Temperature; cooling dynamics	Reagent-free continuous readout	Radiation, airflow, placement	Water-response screening	[67]

Table 3 is a qualitative synthesis, not a common-condition benchmark. Thermal, strain, and impedance devices favor frequent readout but require environmental and attachment compensation. Electrochemical selectivity adds reference, fouling, and calibration demands; analyte specificity is not crop-state specificity. Optical requirements depend on illumination, interrogation, and surface condition. Acoustic emissions need source validation; the cited QCM assay instead measures pathogen DNA in prepared samples. No universal winner is established. Select by the target state, duration, and maintenance capacity, comparing calibrated error, valid-data yield, drift, and plant disturbance over matched periods.

## 3. Plant Microenvironment and Growth Sensing for Crop-State Assessment

### 3.1. Temperature and Humidity Sensing for Plant Water Status Assessment

Temperature and humidity strongly influence plant water relations, photosynthesis, respiration, stomatal behavior, and nutrient transport, and their combined effects ultimately shape crop growth and productivity. In particular, leaf temperature reflects the balance between environmental heating and transpirational cooling, whereas humidity near the leaf surface affects vapor-pressure gradients and stomatal water loss. Continuous monitoring of these parameters therefore provides important information for evaluating plant water status and environmental stress in precision agriculture [68,69].

Conventional agricultural temperature monitoring primarily relies on thermistors and thermocouples. These devices directly measure temperature and offer high sensitivity, low cost, and broad measurement ranges. Thermocouples require cold-junction compensation and amplification of small voltage signals; thermistors instead require resistance calibration and control of self-heating. In contrast, humidity cannot be measured directly and must be inferred from the adsorption of water molecules by humidity-sensitive materials and the resulting physicochemical changes. This indirect process is susceptible to temperature cross-sensitivity, material aging, and contamination, leading to limited stability and frequent calibration requirements. Capacitive humidity sensors detect changes in the dielectric constant of humidity-sensitive media and generally exhibit good linearity and stability. Resistive humidity sensors operate through resistance variations, often involving the Grotthuss proton-hopping mechanism, and are characterized by rapid response and simple structures. Optical fiber humidity sensors use light as the signal carrier and offer immunity to electromagnetic interference and passive operation. Their performance, however, remains constrained by the availability of suitable humidity-sensitive materials and effective temperature-compensation strategies [70,71,72].

Although these technologies effectively characterize environmental temperature and humidity variations, environmental parameters do not necessarily reflect the actual physiological status of plants. With advances in plant phenotyping and precision agriculture, researchers have increasingly recognized that surface microclimatic measurements can resolve some plant responses to environmental fluctuations. Leaf temperature results from the combined effects of stomatal conductance, transpiration, and energy balance, whereas leaf-surface humidity is directly associated with stomatal regulation, water transport, and transpiration processes. Therefore, compared with environmental-scale measurements alone, direct monitoring of temperature and humidity at the plant surface can provide information more closely associated with local plant–atmosphere exchange and physiological responses to external conditions [73,74,75,76].

**Figure 7:**
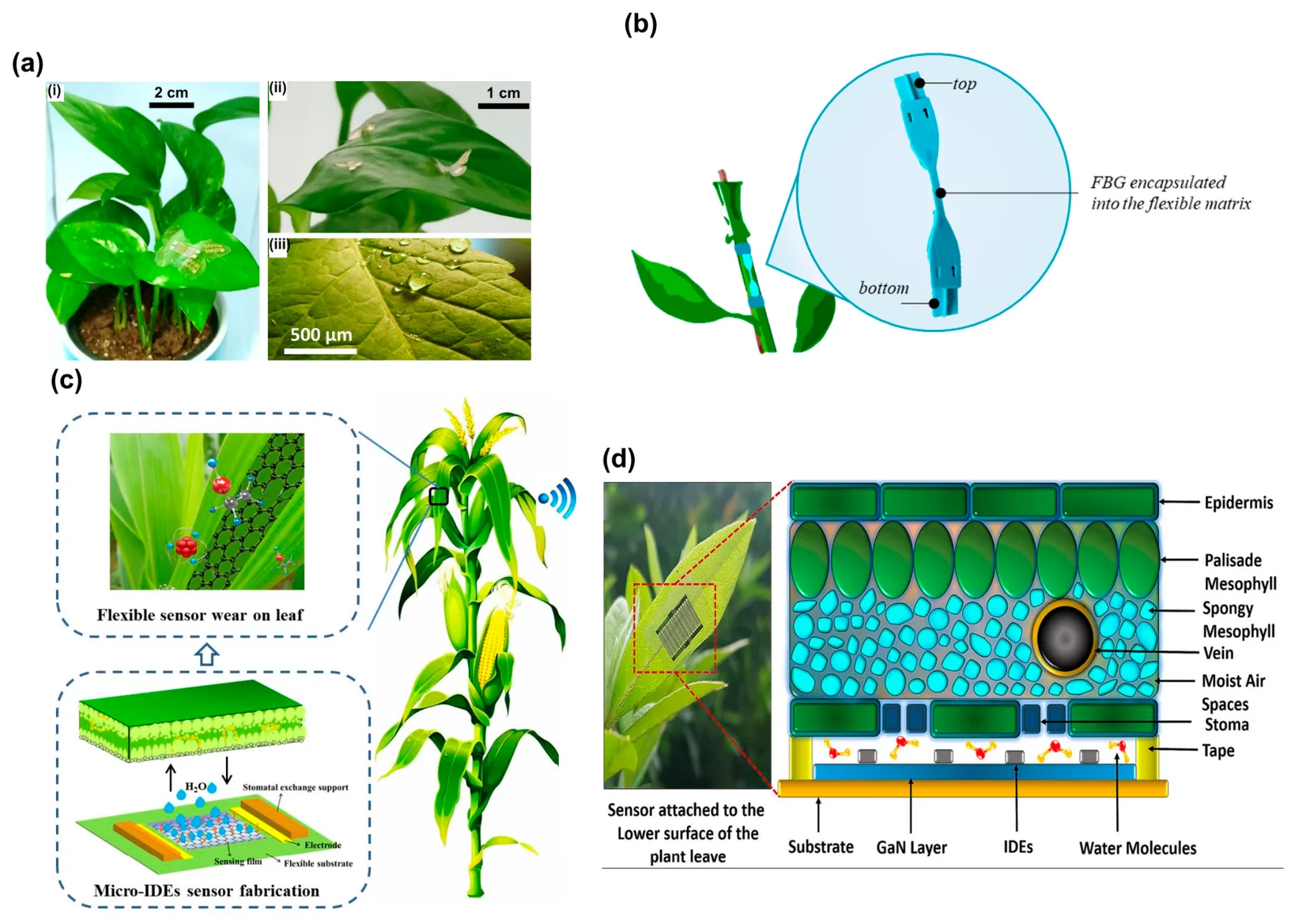
Wearable temperature and humidity sensors for plant monitoring. (**a**) Compliant plant wearable sensor for localized microclimate monitoring [77]. (**b**) Chitosan-coated fiber Bragg grating (FBG) humidity sensor [78]. (**c**) Graphene oxide-based wearable humidity sensor for plant water monitoring [82]. (**d**) Gallium nitride (GaN)-based leaf-mounted humidity sensor for real-time monitoring of plant transpiration [86]. In (**a**), internal labels (**i**–**iii**) show the device on a plant, conformal leaf attachment, and the leaf surface at higher magnification. Panel (**d**) shows the leaf-mounted device and a cross-sectional schematic of humidity sensing beneath the leaf. Panels (**a**–**d**) are adapted from the respective sources cited above under the terms of the Creative Commons Attribution 4.0 International License (CC BY 4.0).

Early studies on plant wearable temperature and humidity sensors focused primarily on achieving stable attachment to plant surfaces and enabling continuous monitoring. With the development of flexible sensing materials and conformal device architectures, increasing attention has been directed toward maintaining reliable sensor–plant contact while minimizing interference with normal plant growth. Nassar et al. [77] developed compliant plant wearable sensors for localized temperature, humidity, and growth monitoring, demonstrating the feasibility of conformal sensor integration with plant surfaces (Figure 7a). To address plant microclimate monitoring requirements, Lo Presti et al. [78] developed FBG-based wearable sensors for temperature and relative-humidity monitoring, including a chitosan-coated FBG humidity sensor (Figure 7b). Their leaf-mounted device employed a porous PDMS substrate.

In parallel, increasing attention was directed toward improving sensor conformability and breathability to accommodate complex plant surface structures and continuous growth. Wei et al. [79] developed a wearable humidity sensor consisting of a graphene oxide (GO) humidity-sensitive layer supported by a porous polytetrafluoroethylene substrate. The porous architecture improved both device breathability and adaptation to leaf-surface microtopography, enabling stable contact and continuous humidity monitoring on plant surfaces. Breathable structures can reduce occlusion and accommodate growth, but their effects on stomatal exchange and attachment stability must be measured [80].

Advances in flexible interface engineering and device integration have gradually propelled plant wearable temperature and humidity sensors from laboratory validation toward field applications. Yin et al. [81] integrated a graphene-based humidity-sensitive element with a gold-film thermistor on a PI substrate, enabling continuous monitoring of temperature and humidity on maize leaves for more than two weeks and supporting extended on-plant operation over the tested interval, rather than establishing season-long reliability. Li et al. [82] further developed a flexible nano-graphene oxide-based wearable sensor for noninvasive real-time monitoring of leaf transpiration and plant water dynamics (Figure 7c). Subsequently, efforts shifted toward improving device integration and intelligence. Huang et al. [83] employed a covalent organic framework film as the humidity-sensitive layer and combined it with a thermistor and wireless communication module to construct a wearable monitoring system capable of simultaneous acquisition and wireless transmission of temperature and humidity signals from tomato leaves. These advances in long-term stability, multiparameter integration, and wireless readout have shifted attention from simply recording the plant microclimate toward interpreting the physiological processes reflected by these signals [84].

Accordingly, the role of plant-wearable temperature and humidity sensing is expanding from microclimate measurement toward physiological-state assessment. Leaf temperature and leaf-surface humidity fundamentally reflect transpiration, water transport, and stomatal regulation, making their continuous monitoring valuable for plant-state assessment. Li et al. [85] developed a flexible wearable sensor capable of simultaneously measuring leaf temperature, humidity, and leaf-scale vapor pressure deficit. These measurements enabled nondestructive in situ monitoring of leaf transpiration, demonstrating the transition of wearable temperature and humidity sensors from environmental sensing tools to platforms for physiological state analysis. Furqan et al. [86] further demonstrated a gallium nitride (GaN)-based capacitive humidity sensor directly attached to the lower surface of plant leaves for real-time transpiration monitoring (Figure 7d). The sensor tracked transpiration-related humidity changes over five days as soil water availability decreased, demonstrating the feasibility of using leaf-mounted humidity sensing to follow plant water dynamics over extended periods. Dynamic tracking of these microclimatic parameters can further provide indirect information on variations in transpiration, stomatal regulation, and plant water response, thereby supporting plant phenotyping, stress-response analysis, and precision agricultural management [87,88].

The studies summarized in Table 4 illustrate a shift from passive microclimate recording toward physiological interpretation of crop water status. Continuous measurements of leaf temperature, surface humidity, and leaf-scale vapor pressure deficit capture time-resolved changes associated with transpiration, stomatal regulation, and plant water demand. Combined with soil-moisture, meteorological, and canopy-temperature data, these measurements can help distinguish environmental forcing from plant-specific water responses and improve assessment of spatial and temporal variation in crop water status [89]. Time-series analysis can further identify persistent deviations associated with water stress and quantify recovery after irrigation. Thus, the principal value of plant-wearable temperature and humidity sensing lies not simply in measuring the local microclimate, but in relating these measurements to crop water demand and irrigation response.

**Table 4:** Wearable Temperature and Humidity Sensors for Microclimate Monitoring.

Target	Technique	Features	Application	Reference
Temperature and humidity	Au on PI	Low power; flexible	Microclimate monitoring	[77]
Temperature and humidity	ZnIn_2_S_4_	Stable; fast response	Light stress monitoring	[90]
Humidity	LIG/GO on PI	High sensitivity; low hysteresis	Leaf transpiration monitoring	[91]
Temperature and humidity	Au/LIG on PI	Multipoint monitoring	Field VPD monitoring	[81]
Temperature	Au on PET	Adhesive-free	Physiological monitoring	[92]

Abbreviations: polyimide (PI); laser-induced graphene (LIG); graphene oxide (GO); vapor pressure deficit (VPD); polyethylene terephthalate (PET).

### 3.2. Growth Strain Sensing for Water Status and Growth Dynamics

Growth-induced strain provides a direct physical indicator of crop growth and water-related responses, including stem expansion and contraction, fruit enlargement, and leaf extension. These deformation patterns are strongly influenced by the balance between transpiration and root water uptake, producing characteristic diurnal cycles associated with plant water status and growth vigor. Manual calipers and tapes provide intermittent observations, whereas instrumented displacement sensors can record continuous growth. Wearable devices seek to reduce the size and attachment burden of continuous measurements. Wearable strain sensors address this limitation by continuously converting subtle tissue deformation into quantifiable signals. Their practical development, however, requires simultaneous optimization of sensitivity, mechanical compliance, and long-term plant–sensor interface stability, because growing tissues continuously change in size, curvature, and surface properties.

**Figure 8:**
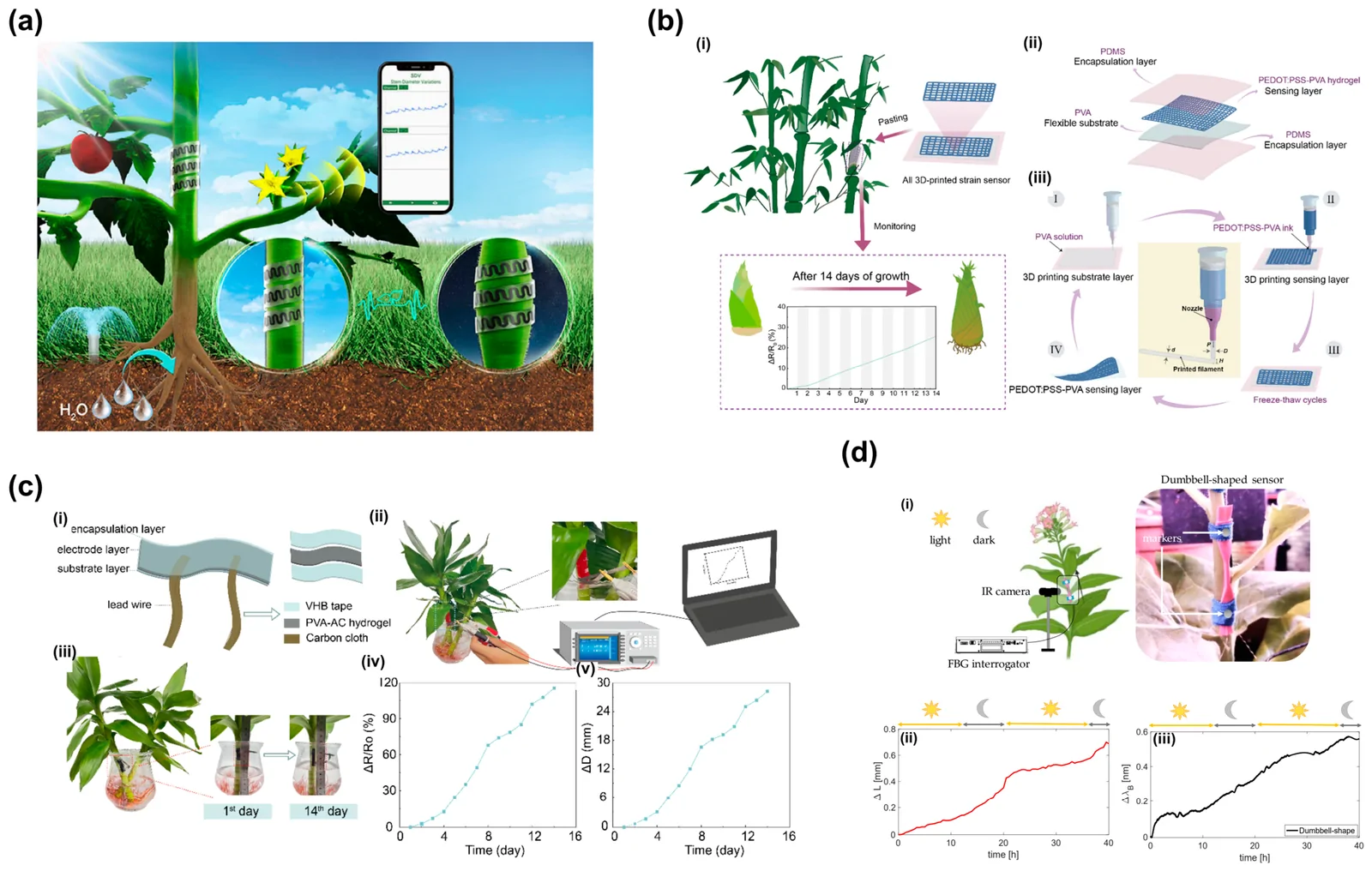
Wearable strain sensors for plant growth monitoring. (**a**) Biomimetic wearable sensor for in vivo plant pulse monitoring [93]. (**b**) 3D-printed adaptive hydrogel strain sensor for plant growth monitoring [97]. (**c**) Polyvinyl alcohol/activated charcoal hydrogel strain sensor for long-term stable plant growth monitoring [96]. (**d**) Fiber Bragg grating (FBG) strain sensor encapsulated with Dragon Skin silicone for stem and fruit growth monitoring [94]. In (**b**), (**i**–**iii**) show growth monitoring, the multilayer device structure, and fabrication; steps I–IV denote substrate printing, sensing-layer printing, freeze–thaw processing, and the resulting sensing layer. In (**c**), (**i**–**v**) show device construction, the monitoring setup, attachment on days 1 and 14, relative resistance change, and inferred diameter change. In (**d**), (**i**–**iii**) show the experimental setup, reference elongation, and temperature-compensated Bragg-wavelength shift. Light and dark symbols identify illumination periods. Panels (**a**–**d**) are adapted from the respective sources cited above under the terms of the Creative Commons Attribution 4.0 International License (CC BY 4.0).

Efforts to accurately capture subtle deformations initially focused on improving sensor sensitivity and detection range. Zhang et al. [93] developed a biomimetic adaptive winding strain sensor inspired by plant tendrils, enabling conformal attachment to tomato stems and continuous monitoring of subtle stem expansion and contraction associated with plant pulse (Figure 8a). Nevertheless, such electrical sensors remain susceptible to environmental interference, and their long-term monitoring stability under complex field conditions remains challenging. To improve signal reliability, researchers subsequently explored optical signal-transmission strategies for strain monitoring.

FBG sensors have emerged as a promising solution for long-term stable monitoring because of their high sensitivity, chemical inertness, and passive operation at the sensing element, although the interrogator requires power. Lo Presti et al. [94] optimized FBG sensors through silicone-based flexible encapsulation and conformal structural design. For stem monitoring, a dumbbell-shaped structure concentrated strain within the grating region, achieving a strain sensitivity of 0.04 nm·mε^−1^ (Figure 8d). For fruit monitoring, a ring-shaped structure encapsulated the grating off-axis to detect bending strain, yielding a radius-change sensitivity of 0.04 nm·mm^−1^. This system was successfully applied to monitor tobacco stem elongation and melon fruit expansion and distinguished fruit size changes caused by water transport from those associated with growth during a 60 h field experiment. Although FBG sensors offer good signal stability, the mechanical stiffness and limited extensibility of conventional optical fibers can constrain their use on rapidly growing or highly deformable plant organs. This limitation has encouraged the development of softer and more stretchable sensing materials for long-term growth monitoring.

Emerging flexible materials, particularly hydrogels and conductive polymers, have recently become a major focus in plant strain monitoring. Owing to their excellent hydrophilicity and mechanical compliance, hydrogel sensors can conform closely to plant surfaces and deform synchronously with tissue growth [95]. In addition to environmental stability, low hysteresis and fatigue resistance are essential for reliable long-term growth monitoring. Wang et al. [96] developed a polyvinyl alcohol (PVA)/activated charcoal conductive hydrogel strain sensor with low hysteresis and fatigue resistance, enabling stable monitoring of plant growth over 14 days (Figure 8c). Building on these material-level improvements, Wang et al. [97] employed multimaterial 3D printing to fabricate an integrated multilayer hydrogel strain sensor with high sensitivity and stretchability for accurate plant growth monitoring (Figure 8b), illustrating how integrated fabrication can further improve device consistency and structural adaptability.

Representative systems are summarized in Table 5. Unlike periodic imaging or manual phenotyping, wearable strain sensors can resolve diurnal expansion–contraction cycles and intermittent growth pulses, providing time-resolved information on tissue development, water transport, and growth recovery. Analysis of these temporal patterns can help distinguish normal circadian variation from sustained deviations associated with water limitation or reduced growth vigor [98]. Comparison with canopy, soil, and meteorological observations can then determine whether spatially observed field differences are accompanied by persistent changes in plant growth. However, irreversible growth and reversible hydration both contribute to deformation. Their separation requires diurnal baselines and independent water-status measurements, particularly during rapid development. A 60 h optical-fiber demonstration and a 14-day hydrogel demonstration also test different durations and interfaces; neither alone establishes comparative seasonal durability.

**Table 5:** Wearable Strain Sensors for Plant Growth Monitoring.

Target	Technique	Features	Application	Reference
Eggplant, zucchini	GM/CNTM	High sensitivity; nanometer-level resolution	Fruit circadian growth (20 s/10 s)	[99]
Tobacco stem, melon	FBG/Dragon Skin	Fiber-optic multiplexing; ring-shaped design	Stem elongation (0.017 mm h^−1^); fruit circumference monitoring	[94]
Tomato stem and leaf, cucumber cotyledon/fruit	PEDOT:PSS/SEBS	Photostable; low modulus	Diurnal growth monitoring; youngest cotyledon tracking	[100]
Strawberry fruit	CMC/PAM/Gly-Ca^2+^	Dehydration-resistant; breathable	Wireless monitoring; ideal vs. stress conditions	[101]
Eggplant fruit	Ecoflex/MWCNTs	Zero Poisson’s ratio; low stiffness	Radial diurnal growth monitoring over 10 days	[102]

Abbreviations: graphite membrane (GM); carbon nanotube membrane (CNTM); fiber Bragg grating (FBG); poly(3,4-ethylenedioxythiophene):polystyrene sulfonate (PEDOT:PSS); styrene–ethylene–butylene–styrene (SEBS); carboxymethyl cellulose (CMC); polyacrylamide (PAM); glycerol (Gly); multi-walled carbon nanotubes (MWCNTs).

### 3.3. Gas Sensing at the Plant–Environment Interface

Gaseous compounds around plants provide information on both environmental exposure and plant-associated physiological processes. Agriculturally relevant gases such as CO_2_, NO_2_, NH_3_, and H_2_S can originate from soil, fertilizer application, machinery, atmospheric pollution, or plant metabolism, and their local concentrations may influence crop growth and stress responses [103,104]. Conventional gas sensors based on optical, electrochemical, and related mechanisms enable continuous monitoring of these compounds. However, measurements made at the greenhouse or field scale do not necessarily represent the gaseous microenvironment directly experienced by individual leaves, creating a need for sensing approaches positioned closer to the plant–atmosphere interface [105,106].

**Figure 9:**
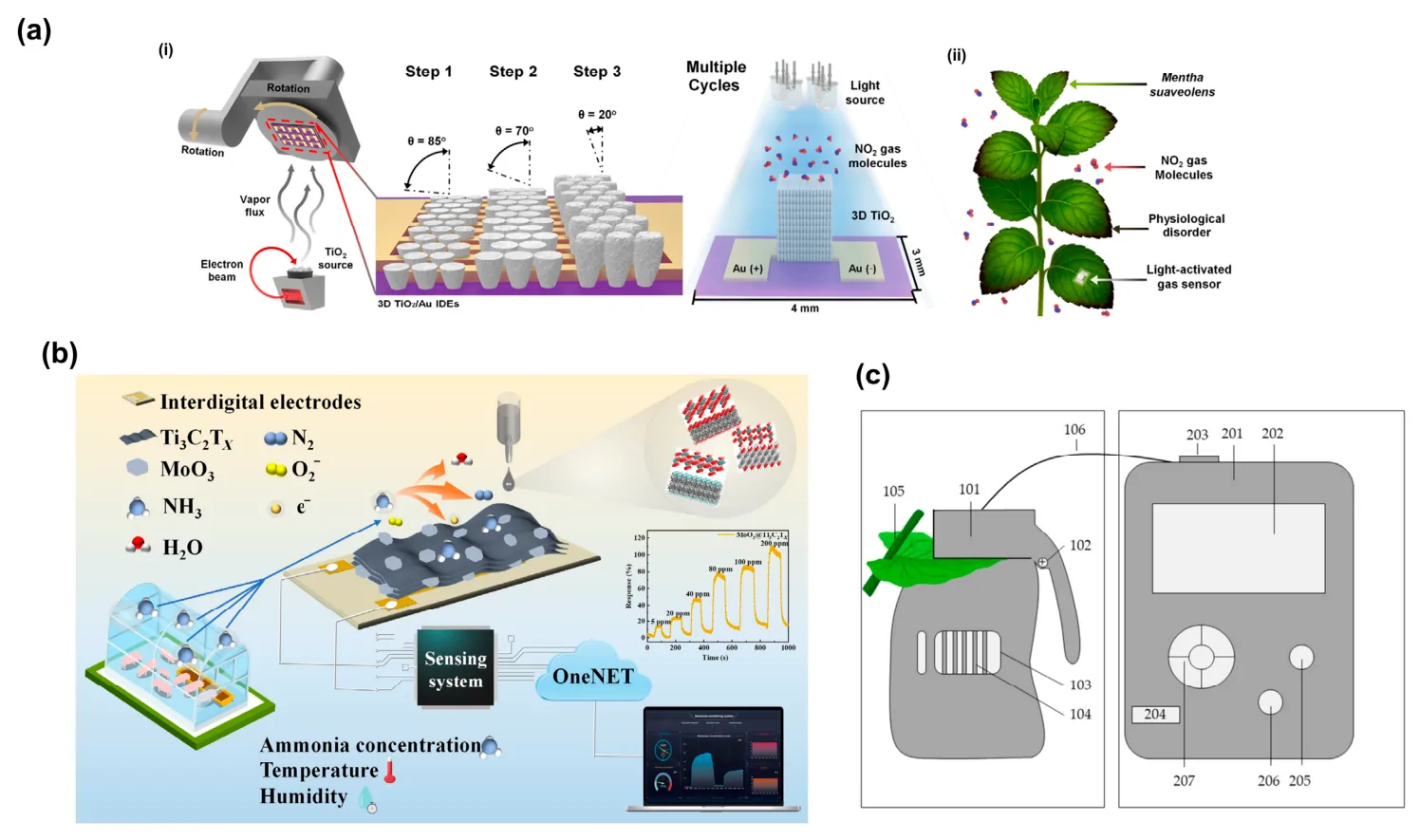
Gas-sensing platforms for agricultural and plant monitoring. (**a**) Photoactive 3D TiO_2_-based NO_2_ sensor for plant health monitoring [114]. (**b**) MoO_3_@Ti_3_C_2_T_x_-based NH_3_ sensor integrated with an intelligent agricultural monitoring system [115]. (**c**) Handheld system for measuring photosynthetic CO_2_ assimilation in plant leaves [107]. In (**a**), (**i**) shows sensor fabrication and (**ii**) shows on-leaf deployment. In (**c**), numbers identify the chamber housing (101), spring clip (102), illuminator window (103), illuminator radiator (104), leaf position (105), connecting cable (106), main-module housing (201), display (202), memory-card module (203), power button (204), recording button (205), potentiometer control (206), and navigation buttons (207). Panels (**a**–**c**) are adapted from the respective sources cited above under the terms of the Creative Commons Attribution 4.0 International License (CC BY 4.0).

As the primary substrate for photosynthesis, CO_2_ is relevant not only to the surrounding gaseous environment but also to plant carbon assimilation when its exchange at the leaf surface is quantified. Conventional infrared gas analyzers can accurately quantify photosynthetic CO_2_ exchange, but their cost, complexity, and dependence on controlled airflow can limit large-scale deployment. Kozlova et al. [107] developed a compact handheld system that estimates leaf photosynthetic CO_2_ assimilation from accumulative changes in CO_2_ concentration within a small measurement chamber (Figure 9c). The system provided measurements consistent with a commercial gas analyzer and detected reductions in assimilation under drought stress, illustrating how localized CO_2_ measurements can be translated into physiological crop-state information [108,109,110,111,112].

A different requirement arises when gaseous exposure must be resolved directly at the plant surface. Measurements made at the greenhouse or field scale may not represent the gaseous conditions experienced by individual leaves, while high-spatial-resolution monitoring near stomatal or boundary-layer regions requires sensors that operate close to the plant–atmosphere interface. Such devices must maintain sensing performance under bending, high humidity, and continuous plant motion. Earlier work demonstrated that biomimetic adhesive architectures could maintain stable leaf attachment while enabling localized NO_2_ detection under bending and humid conditions [113]. More recently, Cho et al. [114] developed a thermally passive NO_2_ sensor based on photoactivated three-dimensional TiO_2_ nanoarchitectures that operates at room temperature under ambient light (Figure 9a). Integration with wireless electronics and a mobile interface enabled autonomous NO_2_ monitoring on *Mentha suaveolens* and real-time tracking of gas-induced physiological stress.

Related intelligent-agriculture gas platforms illustrate how localized gas detection can also be incorporated into connected monitoring systems, although not all such systems are directly plant-wearable. Li et al. [115] constructed a room-temperature MoO_3_@Ti_3_C_2_T_x_ MXene sensor and integrated it into a multifunctional Internet of Things (IoT) monitoring system for intelligent agriculture (Figure 9b). The platform combined sensitive NH_3_ detection with real-time visualization and threshold-triggered warning, demonstrating the progression from standalone gas sensing toward networked agricultural monitoring.

The complex gaseous environment encountered under field conditions imposes stringent requirements on sensor selectivity. Although plant-interfaced sensors can detect individual gases such as NO_2_ or NH_3_, cross-reactivity remains a major limitation in multigas environments. Electronic-nose systems address this problem by combining cross-sensitive sensor arrays with pattern-recognition algorithms. Chu et al. [116], for example, coupled a metal oxide semiconductor sensor array with neural-network analysis to distinguish different NO_2_/CO mixtures, demonstrating the value of algorithm-assisted multigas identification. Transferring such array-based recognition strategies to flexible plant-interfaced devices could enable localized discrimination of complex gaseous signals at the plant–atmosphere interface. However, this transition remains at an early stage because long-term plant attachment, humidity tolerance, cross-gas selectivity, low-power operation, and physiological non-interference must be achieved simultaneously.

Translating these capabilities into flexible plant wearable platforms could enable localized and continuous monitoring of complex gaseous signals directly at the plant–atmosphere interface. However, plant-interfaced gas-sensing systems remain relatively scarce, particularly for long-term monitoring of multiple gases and direct quantification of plant gas-exchange processes. Their practical development is constrained by the need to maintain conformal attachment, minimize humidity and cross-gas interference, reduce power consumption, and preserve stable sensing performance without disturbing plant physiology [117,118]. Representative wearable gas-sensing platforms are summarized in Table 6.

Overall, gas sensing at the plant–environment interface has fewer established deployment pathways than simple temperature or strain monitoring; its maturity relative to endogenous electrochemical sensing remains target- and device-dependent. Recent platforms demonstrate that gas measurements can provide complementary information on both external gaseous exposure and physiological processes such as photosynthetic CO_2_ assimilation. Flexible gas sensors, plant-interfaced devices, and sensor arrays have expanded localized monitoring capabilities, but practical deployment still depends on improved selectivity, stable plant–sensor attachment, humidity tolerance, low-power operation, and validation under realistic field conditions. Addressing these limitations will be essential for developing plant wearable gas sensors as reliable components of multimodal crop-state monitoring.

**Table 6:** Wearable Gas Sensors for Plant Stress Monitoring.

Target	Technique	Features	Application	Reference
NO_2_	AgNPs/rGO	Adhesive-free spray coating	Leaf monitoring	[119]
NO_2_	MWCNTs/PDMS	Biomimetic adhesion; water-wash resistance	Exhaust monitoring	[120]
H_2_O_2_, H_2_S	AuNS@ZIF-8/4-MPBA	Dual-analyte synchronous detection	Stress diagnosis	[121]

Abbreviations: silver nanoparticles (AgNPs); reduced graphene oxide (rGO); multi-walled carbon nanotubes (MWCNTs); polydimethylsiloxane (PDMS); gold nanostars (AuNS); zeolitic imidazolate framework-8 (ZIF-8); 4-mercaptophenylboronic acid (4-MPBA).

## 4. Endogenous Sensing for Nutrient and Physiological State Assessment

Beyond external environmental conditions and surface-associated physical signals, crop physiological status is also reflected by dynamic changes in internal chemical states. Nutrient ions participate in mineral nutrition and osmotic regulation, endogenous metabolites reflect carbon assimilation and metabolic activity, and phytohormones coordinate processes ranging from stomatal regulation to defense responses. Continuous monitoring of these biochemical signals can therefore provide direct plant-level information on nutrient uptake, physiological regulation, and metabolic responses, supporting more detailed assessment of crop physiological state.

### 4.1. Ion Sensing for Nutrient Status and Salinity Assessment

Ionic composition is closely associated with crop nutrition, salinity responses, and contaminant exposure. Heavy-metal ions such as Cd^2+^ and Pb^2+^ are important targets in agricultural monitoring because they can accumulate in soils and crops and interfere with normal plant physiological processes [122,123,124]. In parallel, nutrient-related ions such as NO_3_^−^, NH_4_^+^, phosphate species, and K^+^ are involved in plant mineral nutrition, whereas excessive accumulation of ions such as Na^+^ is closely associated with salinity stress. In situ monitoring of these ionic signals can therefore provide important information for nutrient-status assessment, salinity diagnosis, and contaminant monitoring in precision agriculture [125,126,127,128].

Conventional ion analysis generally relies on sample collection and laboratory-based instrumentation, limiting its suitability for continuous monitoring of dynamic ionic changes in living plants. Although optical and electrochemical methods have both been explored for rapid ion analysis, electrochemical sensing is particularly compatible with flexible substrates, ion-selective interfaces, microneedles, and transistor architectures. These features facilitate minimally invasive or plant-integrated measurements and provide a practical route toward continuous in situ monitoring of nutrient uptake, salinity responses, and contaminant exposure [129,130].

**Figure 10:**
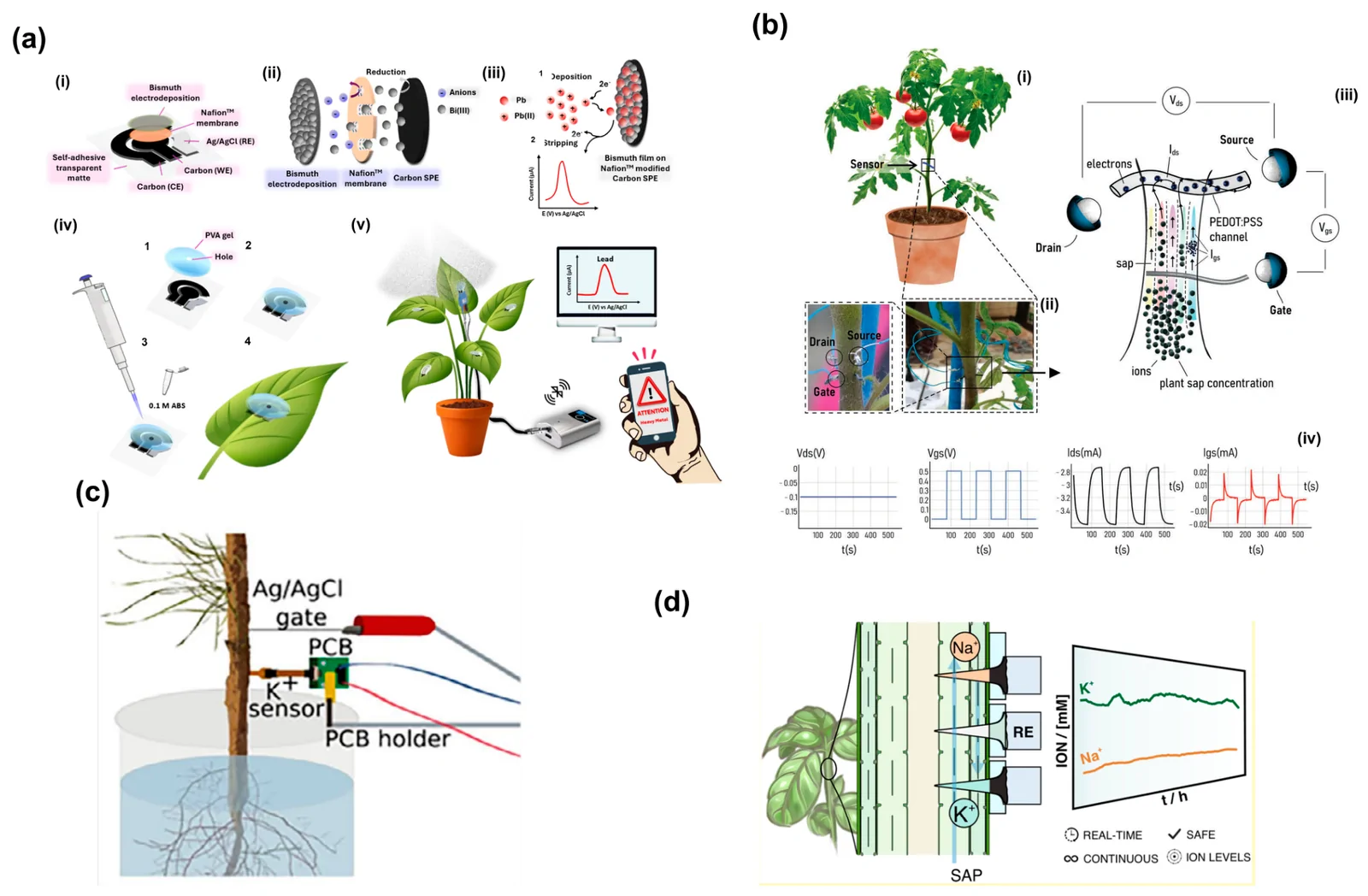
Wearable sensors for plant ion monitoring. (**a**) Plant wearable electrochemical sensor for atmospheric lead detection [133]. (**b**) Organic electrochemical transistor (OECT)-based sensor for ion concentration monitoring in plant sap [140]. (**c**) Implantable ion-selective OECT for continuous in vivo K^+^ monitoring [144]. (**d**) In planta potentiometric microneedle sensor for K^+^ and Na^+^ monitoring [138]. In (**a**), (**i**–**v**) show electrode construction, bismuth deposition and membrane structure, lead deposition/stripping, gel-assisted leaf attachment and electrolyte addition, and wireless monitoring. Steps 1 and 2 in (**iii**) denote deposition and stripping; steps 1–4 in (**iv**) show gel preparation/placement, electrolyte addition, and leaf attachment. In (**b**), (**i**–**iv**) show plant deployment, the stem interface, the transistor sensing principle, and electrical readout. Arrows indicate assembly or reaction steps, charge/ion transport, and signal transmission as depicted; dashed connectors identify enlarged views. Panels (**a**–**d**) are adapted from the respective sources cited above under the terms of the Creative Commons Attribution 4.0 International License (CC BY 4.0).

Heavy-metal monitoring provides an early example of the transition from ex situ ion analysis toward direct assessment of plant exposure. Wang, Yu et al. [131] developed a portable electrochemical platform for detecting heavy-metal ions in crop and soil samples, although the measurements still relied on collected samples [132]. By contrast, Seker et al. [133] developed a self-adhesive electrochemical sensor that could be directly attached to plant leaves for in situ monitoring of atmospheric Pb contamination (Figure 10a). By integrating contaminant collection with electrochemical detection directly on the leaf surface, the device demonstrated the potential of wearable sensing to assess leaf-level exposure to atmospheric Pb rather than environmental contamination alone [134,135,136].

Beyond contaminant exposure, nutrient- and salinity-related ions provide more direct information on internal plant physiological status. Ibrahim, Yin, et al. [137] developed a minimally invasive microneedle electrochemical sensor for direct nitrate analysis in maize stem sap, demonstrating the feasibility of accessing nutrient ions within living tissues. More recently, Wang et al. [138] developed a dual potentiometric microneedle platform for simultaneous K^+^ and Na^+^ monitoring in living plants (Figure 10d). The sensor enabled rapid and continuous ion-selective measurements and captured dynamic changes in K^+^ and Na^+^ concentrations as well as their transport from the nutrient solution into plant stems. This capability extends in planta ion sensing from discrete concentration measurements toward time-resolved characterization of nutrient transport and ionic responses [139].

Beyond conventional potentiometric ion sensing, transistor-based architectures provide an alternative route for continuously coupling ionic changes with plant physiological processes. Gentile et al. [140] developed a biomimetic and biocompatible organic electrochemical transistor (OECT) sensor by directly implanting poly(3,4-ethylenedioxythiophene):polystyrene sulfonate (PEDOT:PSS)-functionalized fiber electrodes into tomato stems. The device utilized native plant sap as the electrolyte and monitored variations in gate and channel currents to simultaneously quantify total ion concentration and xylem water saturation during continuous in vivo monitoring (Figure 10b). This approach demonstrated that ion-related measurements can be interpreted together with plant water status rather than as isolated concentration values [141,142,143].

More recently, OECT architectures have also been adapted for ion-specific monitoring. Han et al. [144] developed an implantable ion-selective OECT for continuous K^+^ monitoring directly within plant xylem (Figure 10c). The ion-selective interface enabled stable long-term in vivo monitoring, extending OECT-based sensing from overall ionic conditions toward ion-specific physiological tracking. By coupling sensor outputs with a mathematical model, the measured signals were decoupled into independent physiological variables. The system was applied to long-term monitoring of tomato plants under vapor-pressure-deficit, drought, and salinity conditions, with the resulting signals closely corresponding to physiological responses such as stomatal regulation and photosynthetic activity [145].

Collectively, these developments mark a transition from measuring ion availability outside the plant toward directly tracking ion uptake, transport, and accumulation at plant surfaces and within living tissues (Table 7). In planta measurements of K^+^, Na^+^, NO_3_^−^, total ion concentration, and related variables can reveal crop responses to nutrient and salinity conditions, whereas on-plant heavy-metal sensing provides direct evidence of contaminant exposure. Integration with soil, irrigation-water, and other field measurements can further distinguish external ion availability from the corresponding plant response and improve nutrient-status, salinity, and contamination assessment [146,147]. Ion activity or concentration is not equivalent to uptake flux or whole-plant nutrient sufficiency. Sap flow, dilution, competing ions, and electrode reference stability can change the signal independently of nutrient supply. Joint water-status measurements and tissue assays are therefore needed before these dynamics can justify fertilizer or salinity-management thresholds.

**Table 7:** Wearable Ion-Selective Sensors for Plant Nutrition and Contaminant Monitoring.

Target	Technique	Features	Application	Reference
Ions	PEDOT:PSS OECT	High sensitivity	Tomato (drought/salt stress)	[140]
NO_3_^−^	VB12/GO/SU-8	Reductive response	Maize stalk sap	[137]
Na^+^	PEDOT:PSS microneedle	Potentiometric sensing	In planta Na^+^ monitoring	[49]
Pb^2+^	Bi/Nafion/PVA	SWASV method	Atmospheric Pb on leaves	[133]

Abbreviations: poly(3,4-ethylenedioxythiophene):polystyrene sulfonate (PEDOT:PSS); organic electrochemical transistor (OECT); vitamin B12 (VB12); graphene oxide (GO); polyvinyl alcohol (PVA); square-wave anodic stripping voltammetry (SWASV). SU-8 is an epoxy-based photoresist.

### 4.2. Phytohormone Sensing for Stress Interpretation

Plant hormones function as endogenous signaling molecules that regulate a wide range of physiological processes throughout the crop life cycle, from seed germination to stress adaptation, despite being present at extremely low concentrations. Among them, indole-3-acetic acid (IAA), abscisic acid (ABA), ethylene, jasmonic acid (JA), and salicylic acid (SA) participate in growth regulation and stress adaptation, and their dynamic changes can provide important chemical information on crop physiological and stress states [148,149].

Conventional hormone analysis generally requires tissue collection, extraction, and laboratory-based instrumentation, providing discrete measurements rather than continuous time-series data. This limits the ability to resolve rapid hormonal dynamics during stress responses and motivates the development of in situ sensing approaches capable of repeated or continuous measurements in living plants [150]. Early electronic approaches to in situ hormone sensing also faced substantial selectivity and environmental-stability challenges. For gaseous hormones such as ethylene, metal oxide semiconductor (MOS) chemiresistors can provide sensitive detection, but their responses may be affected by humidity, temperature, and interfering gases, complicating reliable long-term interpretation under field conditions [151]. These limitations have encouraged the development of more selective recognition interfaces and plant-compatible wearable configurations [152,153].

**Figure 11:**
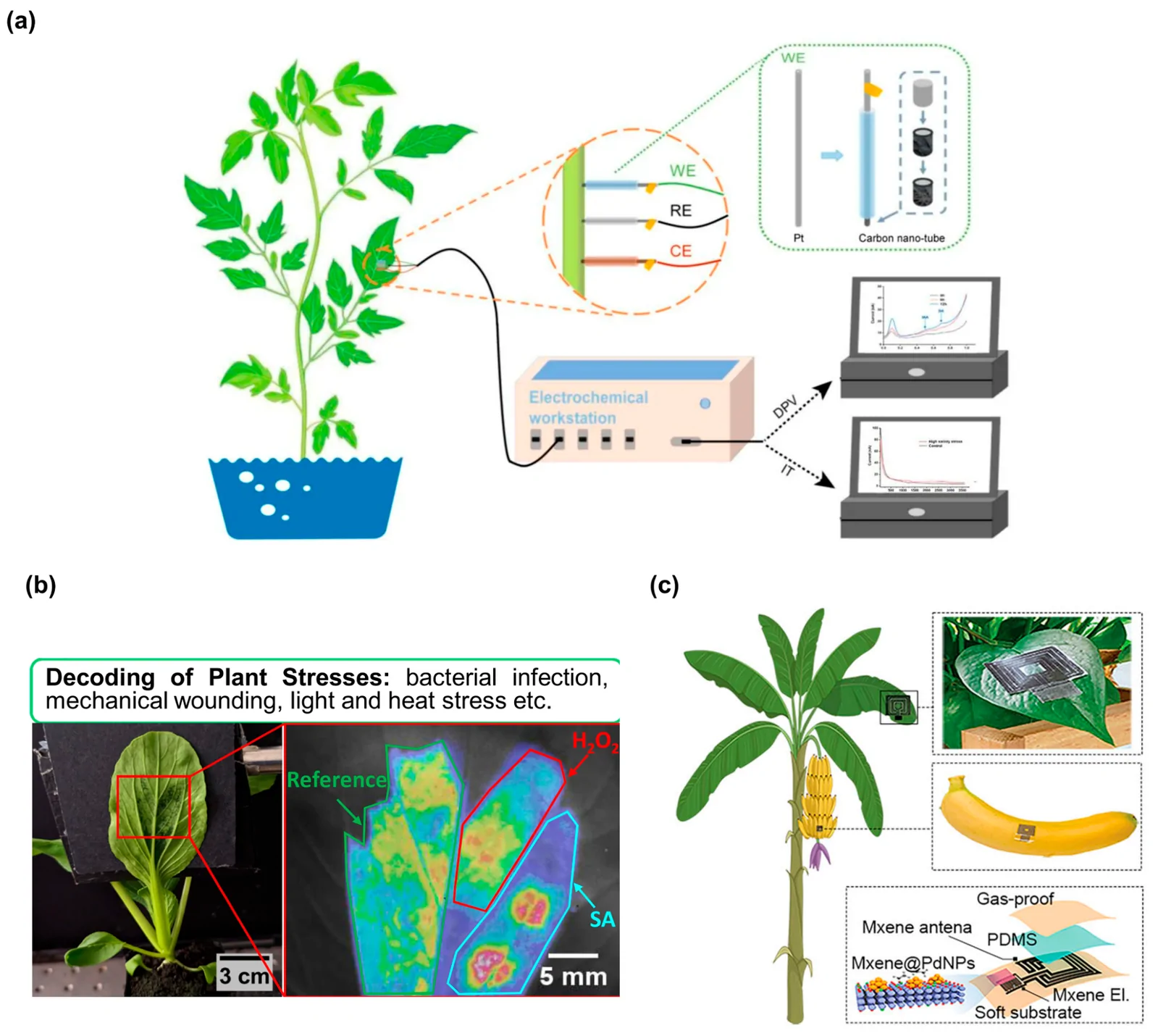
In vivo and wearable sensing strategies for plant hormone monitoring. (**a**) Electrochemical microsensor for continuous in vivo monitoring of indole-3-acetic acid (IAA) and salicylic acid (SA) in tomato leaf veins [155]. (**b**) In planta SA nanosensor multiplexed with hydrogen peroxide (H_2_O_2_) and reference nanosensors for decoding stress-signaling dynamics [158]. (**c**) MXene-printed wireless plant-wearable sensor for in situ ethylene monitoring [161]. Panel (**a**) labels the working electrode (WE), reference electrode (RE), and counter electrode (CE). In (**a**), DPV denotes differential pulse voltammetry and IT denotes the current–time recording. In (**b**), green, red, and cyan outlines identify reference, H_2_O_2_, and SA nanosensor regions, respectively; false colors visualize their fluorescence patterns. The images illustrate sensor configuration and spatial responses rather than a shared quantitative color scale. Panels (**a**) and (**b**) adapted from Refs. [155,158], respectively, under the terms of the Creative Commons Attribution 4.0 International License (CC BY 4.0). Panel (**c**) adapted from Figure 7a of Ref. [161], which was adapted from Ref. [159] and licensed under CC BY 4.0.

To overcome the limitations of conventional analytical methods in spatiotemporal resolution and field applicability, early in situ hormone sensing focused on miniaturized electrochemical platforms capable of directly accessing plant tissues. Wang et al. [154] developed an Au@SnO_2_/vertical-graphene microneedle sensor for label-free ABA detection and successfully quantified ABA in cucumber, Arabidopsis, and grape tissues, demonstrating the feasibility of implantable electrochemical hormone monitoring. Extending this approach toward simultaneous hormone analysis, Tang et al. [155] developed an electrochemical microsensor for continuous in vivo monitoring of both IAA and SA in tomato leaf veins (Figure 11a). The minimally invasive platform resolved dynamic changes in both hormones under salinity stress, illustrating how continuous multi-hormone measurements can capture coordinated physiological responses that are inaccessible to destructive endpoint analysis.

As in situ hormone sensing progressed, molecular selectivity became a central challenge because structurally related compounds and electroactive interferents coexist in plant fluids. Biomimetic recognition strategies such as molecularly imprinted polymers (MIPs) provide target-specific binding cavities while offering greater environmental robustness than many biological recognition elements. Bukhamsin et al. [53] coated MIPs onto microneedle surfaces for selective SA recognition after insertion into plant tissues. Liu et al. [156] further integrated a flexible Fe-doped graphene SA sensor with a smartphone-controlled electrochemical analyzer, enabling portable real-time monitoring of SA dynamics in salt-stressed cucumber plants. Haridass et al. [157] developed a microneedle molecularly imprinted polymer (MIP) sensor for methyl jasmonate (MeJA) and dynamically monitored endogenous MeJA during fall armyworm feeding in maize, revealing faster and stronger hormone induction in resistant genotypes than in susceptible plants.

Beyond improving single-hormone selectivity, recent platforms increasingly emphasize temporal relationships among multiple signaling molecules. Ang et al. [158] developed an in planta near-infrared nanosensor for SA and multiplexed it with H_2_O_2_ and reference nanosensors (Figure 11b), enabling simultaneous monitoring of stress-induced signaling waves in living plants. Distinct temporal patterns under light, heat, pathogen, and mechanical-wounding stresses showed that coordinated signaling profiles can provide information on stress identity before visible symptoms develop. Gaseous phytohormones require a different sensing architecture because they are released from, rather than sampled within, plant tissues. Li et al. [159] developed all-MXene-printed flexible radio-frequency resonators functionalized with MXene decorated with palladium nanoparticles for wireless ethylene detection and attached the sensor tags directly to leaves and fruit surfaces (Figure 11c). The battery-free platform enabled continuous in situ profiling of ethylene emissions associated with fruit ripening, extending hormone sensing from implanted biochemical measurements toward noninvasive wireless monitoring.

The evolution of the systems listed in Table 8 —from destructive endpoint analysis to continuous, in vivo, multiplexed, and wireless measurements—highlights the growing emphasis on hormone dynamics rather than isolated concentration values [160]. Changes in IAA, ABA, SA, JA/MeJA, and ethylene, together with their temporal relationships with other signaling molecules, may precede visible injury and therefore provide early physiological evidence of drought-, salinity-, pathogen-, or herbivore-related responses. In precision agriculture, such measurements could complement environmental observations, canopy phenotyping, and diagnostic information during stress assessment. Their practical interpretation, however, will require improved selectivity in complex plant matrices and field-calibrated relationships between hormone trajectories, crop developmental stage, and stress severity.

**Table 8:** Wearable Sensors for Plant Hormone Monitoring.

Target	Technique	Features	Application	Reference
IAA	Stainless steel microelectrode	Low cost	Soybean seedlings	[162]
ABA	Au@SnO_2_/vertical graphene	High selectivity	Plant leaves	[154]
SA	MIP/magnetic nanoparticles	Minimally invasive; versatile	Tobacco leaves	[53]
SA	Fe-LIG/flexible patch	Smartphone-readable	Cucumber leaves	[156]
MeJA	MIP/microneedle	Pest-resistance genotype differentiation	Maize leaves	[157]
Ethylene	3D origami/thermal isolation	Thermal isolation design	Chrysanthemum leaves	[163]
Ethylene	Biomimetic copper	Noble-metal-free system	Fruits	[164]

Abbreviations: indole-3-acetic acid (IAA); abscisic acid (ABA); salicylic acid (SA); methyl jasmonate (MeJA); molecularly imprinted polymer (MIP); laser-induced graphene (LIG).

### 4.3. Endogenous Metabolite Sensing for Physiological State Assessment

Plant endogenous metabolites provide molecular-level information on physiological status and can respond to environmental changes before visible phenotypic symptoms appear. Among them, sugars are primary products of photosynthesis and major forms of assimilated-carbon transport between plant organs, so their dynamics reflect carbon assimilation, allocation, and metabolic activity. Continuous in situ monitoring of plant sugars can therefore provide insight into carbon metabolism and stress responses. However, conventional methods require tissue sampling, extraction, and laboratory analysis, preventing real-time tracking of rapid sugar fluctuations induced by photoperiod changes, water stress, or pathogen infection [165].

Optical sugar assays can separate sample chemistry from electrical readout, but reagent-based fluorescence is not label-free and does not by itself establish wearable operation. Bagal-Kestwal et al. [166] immobilized invertase and gold nanoclusters on natural onion epidermal membranes and quantified sucrose through fluorescence quenching induced by sucrose hydrolysis products. The sensor achieved a limit of detection of 2 × 10^−9^ M and a response time of 30 s, demonstrating analytical feasibility on a biological substrate, rather than continuous sensing on a living crop. However, signal acquisition required a benchtop fluorometer, and substrate consistency was difficult to maintain, limiting its applicability for portable field deployment.

Electrochemical transduction provides greater practical advantages in device miniaturization, signal processing, and integration with flexible substrates. Noninvasive wearable electrochemical sensors typically induce the migration of interstitial fluid toward the plant surface through reverse iontophoresis, where sugars are subsequently captured by patch electrodes. Perdomo et al. [50] developed a wearable patch incorporating glucose oxidase (GOx)/Prussian blue/carbon-ink electrodes and a PVA/agarose hydrogel matrix. The device enabled temporal recording of glucose dynamics under stress conditions in multiple crop species, achieving a sensitivity of 22.7 nA/(μM·cm^2^). The study demonstrated continuous surface-access monitoring of crop metabolic responses under the tested conditions. To address enzyme deactivation, Yun et al. [167] integrated potassium-doped Co_3_O_4_ catalysts with screen-printed and clip-shaped microneedle electrodes for non-enzymatic glucose detection. The microneedle device monitored light-associated glucose responses in plant leaves and should be distinguished from noninvasive surface extraction. In reverse-iontophoresis systems, signal intensity depends on trans-epidermal transport, which varies with cuticle properties and environmental conditions. Substantial differences in extraction efficiency among species and leaf positions remain a major obstacle to the development of universally applicable calibration strategies [168].

**Figure 12:**
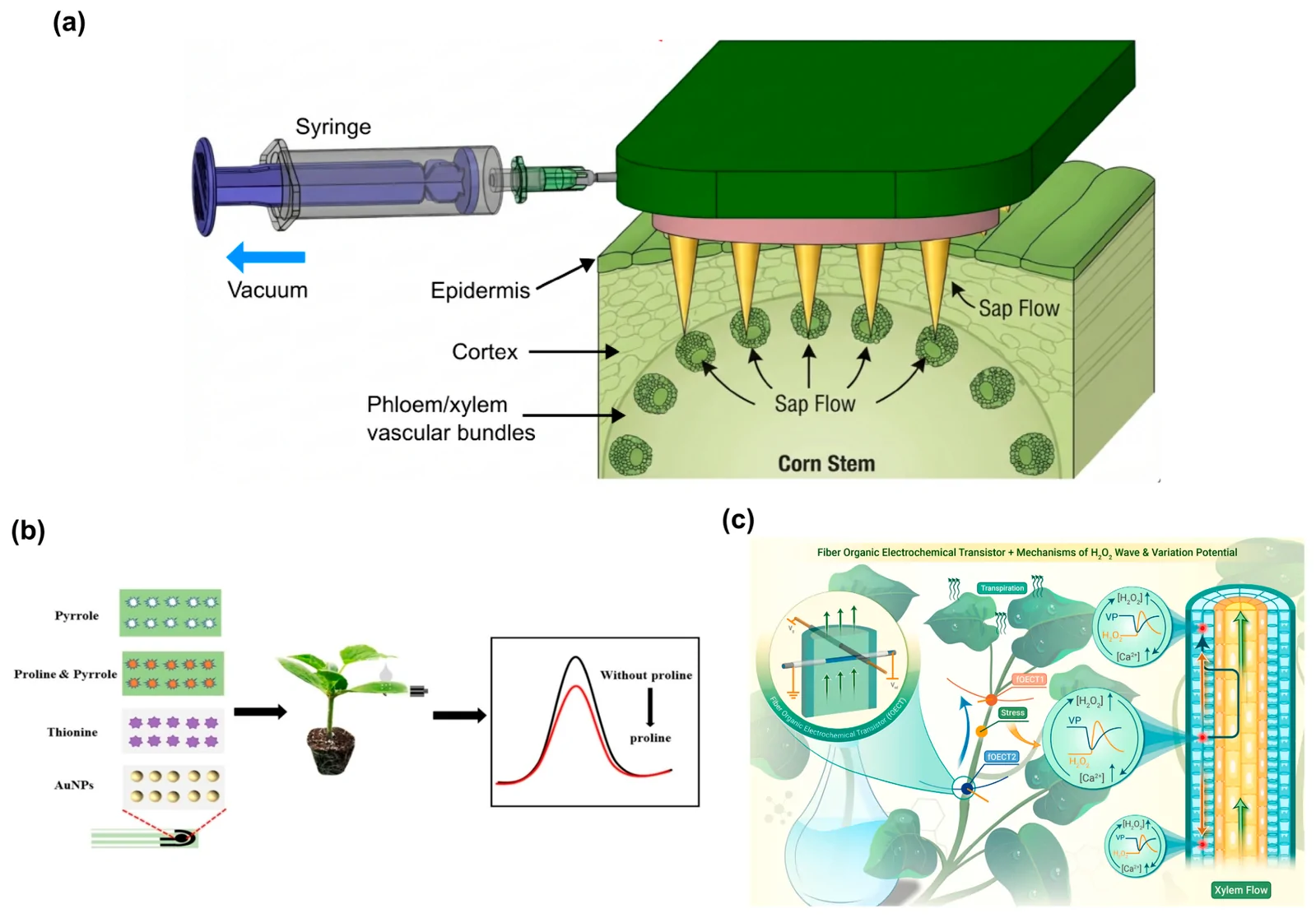
Wearable and in planta electrochemical sensors for endogenous metabolite monitoring. (**a**) Integrated microneedle–microfluidic platform for in planta glucose and water-stress monitoring [170]. (**b**) Molecularly imprinted electrochemical sensor for in situ proline detection [171]. (**c**) Fiber organic electrochemical transistor platform for continuous in planta monitoring of hydrogen peroxide (H_2_O_2_) and variation potential [172]. Panel (**b**) includes gold nanoparticles (AuNPs). In (**a**), the blue arrow denotes vacuum-assisted extraction and black curved arrows indicate sap transport toward the microneedles. In (**b**), arrows connect electrode modification, plant insertion, and electrochemical response. In (**c**), arrows illustrate xylem flow and the depicted propagation of hydrogen peroxide and variation potential (VP); fOECT denotes fiber organic electrochemical transistor. Panels (**a**–**c**) are adapted from the respective sources cited above under the terms of the Creative Commons Attribution 4.0 International License (CC BY 4.0).

Implantable microneedle-based approaches directly access internal sugar concentrations through minimally invasive sampling. Wu et al. [169] developed a multienzyme cascade microneedle sensor for in planta sucrose monitoring, demonstrating continuous tracking of diurnal sugar dynamics in living stems. More recently, Erukainure et al. [170] integrated hollow microneedles with a sealed microfluidic pathway and an electrochemical transducer to enable minimally invasive access to plant sap while physically isolating the sensing electrodes from the insertion site (Figure 12a). These approaches extend wearable sugar sensing from surface-based extraction toward direct access to internal plant fluids, reducing dependence on trans-epidermal transport.

Beyond sugars, stress-responsive endogenous compounds can provide complementary information on plant physiological adjustment. Yan et al. [171] developed a molecularly imprinted electrochemical sensor for in situ detection of free proline in living cucumber leaves (Figure 12b). The MIP recognition layer enabled selective monitoring of proline changes under salt stress, illustrating the potential of synthetic recognition interfaces for tracking endogenous metabolic responses in living plants. Beyond conventional metabolite concentration measurements, continuous monitoring of reactive oxygen species can provide dynamic information on plant stress signaling. Wen et al. [172] developed microfiber-shaped organic electrochemical transistors that could be threaded into living plants for continuous in planta monitoring of H_2_O_2_ and variation potential (Figure 12c), revealing their coupling with transpiration-driven xylem transport during stress responses.

The examples in Table 9 show how wearable and in planta sensing is shifting metabolite analysis from isolated biochemical measurements toward continuous tracking of carbon metabolism and stress-related physiological dynamics. Temporal changes in sugars, proline, H_2_O_2_, and related compounds can indicate whether metabolic responses emerge, persist, or recover as environmental conditions change [173,174]. Linking these trajectories with soil moisture, nutrient availability, meteorological conditions, and canopy observations can improve interpretation of the environmental or management factors associated with the measured response. At present, however, most metabolite signals are better suited to crop-state assessment than to direct irrigation or fertilization control, because robust relationships between individual metabolite trajectories and actionable management thresholds remain insufficiently established [175,176].

**Table 9:** Wearable Sensors for Plant Metabolite Monitoring.

Target	Technique	Features	Application	Reference
Glucose	Reverse-iontophoresis-assisted enzymatic electrochemical biosensor	Non-invasive, continuous	Light/temperature stress warning	[50]
Betaine	MIP electrochemical sensor	High selectivity	Salt stress monitoring	[177]
Gallic acid	Laser-induced graphene flexible electrochemical sensor	High selectivity, Excellent stability	Salt stress response	[178]
H_2_O_2_/Glucose/pH	3D-printed hollow-microneedle electrochemical sensing platform	Simultaneous multi-parameter monitoring	Plant health monitoring	[179]
Sucrose	Plant-insertable multi-enzyme electrochemical biosensor	High sensitivity	Circadian rhythm monitoring	[169]
Glucose	K-doped Co_3_O_4_ microneedle non-enzymatic electrochemical sensor	Enzyme-free, good long-term stability	Diurnal cycle monitoring	[167]

## 5. Wearable Sensing for Early Stress Warning and Precision Crop Protection

Beyond monitoring crop physiological states, plant wearable sensors can also capture stress- and threat-related signals associated with VOC emissions, pathogen infection, and pesticide exposure, thereby extending plant-level sensing toward early stress warning, crop protection, and treatment assessment in precision agriculture.

### 5.1. Stress-Related Volatile Sensing for Early Warning

VOCs are low-molecular-weight organic compounds emitted by plants and microorganisms and can serve as chemical indicators of crop development, stress responses, and postharvest changes. Their composition varies across agricultural systems, with soil and microbial processes generating alcohols, aldehydes, and other volatile compounds, while electronic olfactory systems have increasingly been used to characterize such complex volatile mixtures [180,181,182]. Plants exposed to pests or pathogens release green leaf volatiles and terpenoid defense signals, whereas fruit ripening and spoilage are accompanied by esters, alcohols, and characteristic fungal metabolites. Nondestructive and real-time monitoring of these VOC patterns, particularly when combined with pattern-recognition techniques, can support early stress and disease assessment as well as postharvest quality evaluation [183,184,185].

Conventional VOC analysis primarily relies on laboratory-based techniques such as gas chromatography–mass spectrometry. Offline sampling and analysis can delay interpretation and undersample transient emissions, although headspace collection can be nondestructive and online mass spectrometry can resolve VOC dynamics [186]. VOC sensing research initially addressed these limitations by shifting from laboratory-based offline analysis to field-deployable in situ monitoring. The primary objective of this stage was to eliminate dependence on specialized laboratory facilities and enable real-time acquisition of gaseous signals directly within the plant microenvironment [187,188].

Early field-deployable VOC sensing systems often relied on metal oxide semiconductor (MOS) technology. Although these devices offered improved portability, their practical application was constrained by limited analytical accuracy, relatively high power consumption, and insufficient selectivity. Their elevated operating temperatures hindered long-term deployment in low-power field environments, while cross-sensitivity among multiple gases restricted performance under complex agricultural conditions.

Expansion of sensing-material platforms subsequently broadened the capabilities of wearable VOC monitoring. Functional nanomaterials, including conductive polymers, CNTs, and graphene, are attractive for low-power gas sensing because of their high surface area and favorable electrical properties at room temperature [189]. Ibrahim, Moru et al. [190] combined platinum nanoparticles with conductive-polymer microcrystals to develop a solid-state amperometric methanol sensor, enabling wearable real-time monitoring of methanol emissions from maize leaves. Hossain et al. [191] subsequently constructed a chemiresistive ethylene sensor based on a copper-complex/single-walled carbon nanotube composite that exhibited reversible responses at room temperature, providing a portable route for nondestructive monitoring of fruit ripening. These studies established the feasibility of low-power wearable detection of individual plant-associated volatile compounds, but single-analyte measurements capture only part of the chemical information associated with plant stress.

**Figure 13:**
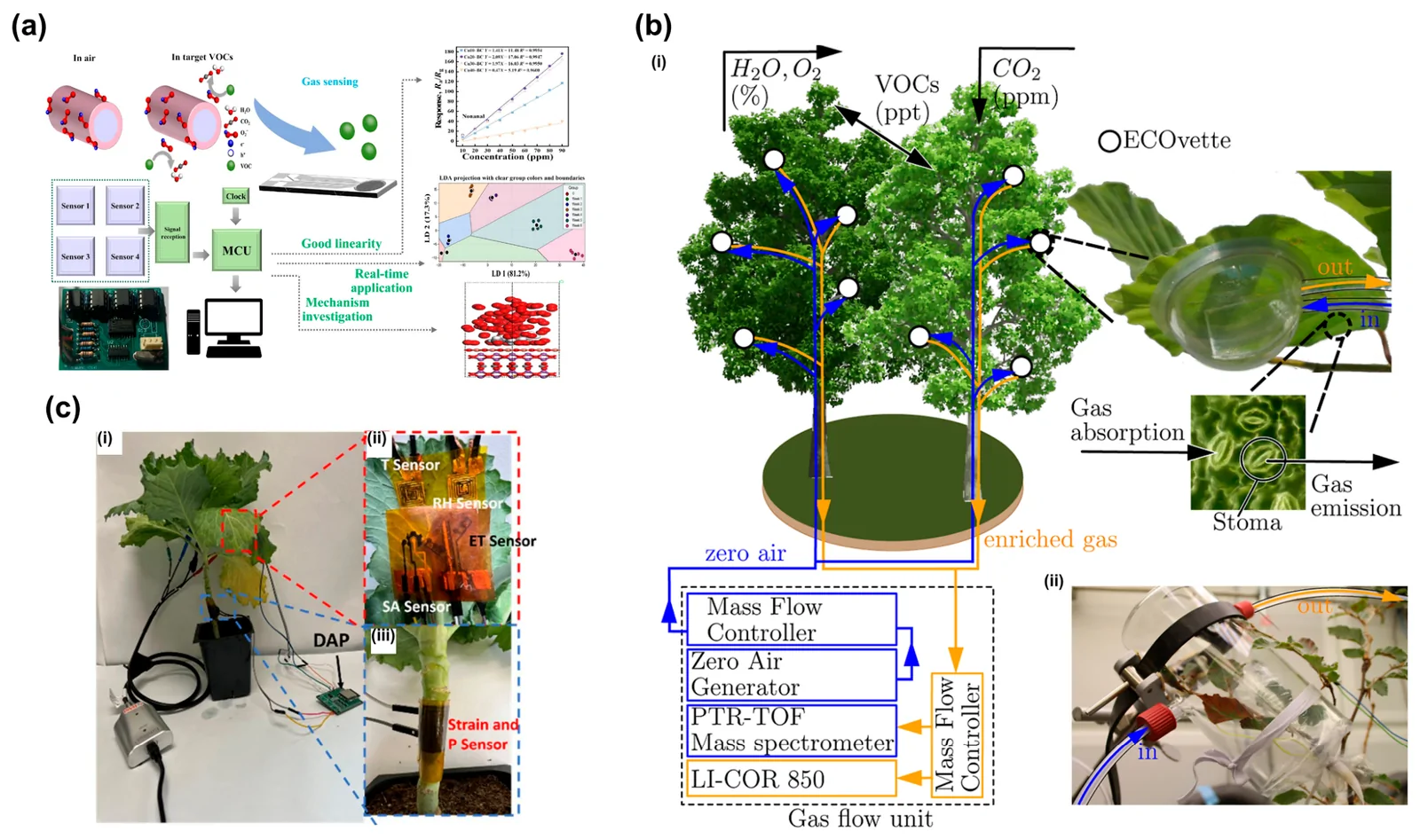
Wearable volatile organic compound (VOC) sensors for plant health monitoring. (**a**) VOC sensor array based on CuO-decorated p–n heterostructured microflowers for volatile compound analysis [196]. (**b**) A wearable system for forest plant gas detection [199]. (**c**) Hybrid multifunctional plant-wearable sensor suite for continuous crop-health monitoring [192]. In (**b**), (**i**) shows the ECOvette measurement network and (**ii**) shows a conventional glass cuvette for comparison; blue and orange arrows indicate incoming zero air and outgoing sample-enriched gas, respectively. In (**c**), (**i**–**iii**) show the complete monitoring setup, leaf-mounted sensors, and stem-mounted strain and pressure sensors. T, RH, ET, SA, P, and DAP denote temperature, relative humidity, ethylene, salicylic acid, pressure, and data acquisition and processing, respectively. Panels (**a**–**c**) are adapted from the respective sources cited above under the terms of the Creative Commons Attribution 4.0 International License (CC BY 4.0).

One route toward richer crop-state information is to combine volatile sensing with other plant-response channels. Hossain and Tabassum [192] developed a hybrid multifunctional physicochemical sensor suite integrating ethylene, salicylic acid, temperature, humidity, strain, and pressure sensing for continuous crop-health monitoring (Figure 13c). Such multimodal platforms broaden the physiological context available for interpreting volatile signals, although they do not by themselves resolve the composition of complex VOC mixtures.

A separate challenge concerns the composition of the volatile signal itself. Stress-induced VOC signatures typically arise from coordinated changes in the relative abundances of multiple compounds rather than from variation in a single analyte. Measurement of individual gas concentrations is therefore often insufficient to capture the multidimensional nature of stress-associated VOC profiles [193,194].

This limitation has driven the integration of cross-reactive sensor arrays with pattern-recognition algorithms. Li et al. [195] combined reduced graphene oxide with gold nanoparticles functionalized by different ligands to construct a wearable sensor array capable of generating cross-reactive responses toward thirteen plant VOCs, marking a transition from quantifying individual gases toward discriminating multicomponent volatile signatures. Zheng et al. [196] further developed a sensor array based on CuO-modified bismuth subcarbonate p–n heterostructure microflowers (Figure 13a) and combined it with pattern-recognition algorithms to classify quality-related VOC profiles. These studies demonstrate how chemical diversity can be encoded as multidimensional response patterns rather than treated as a series of independent single-gas measurements.

The more agriculturally relevant step is to translate this pattern-recognition capability to sensors operating directly on living plants. Lee et al. [197] integrated four chemiresistive sensors together with temperature and humidity sensors on a single flexible substrate and attached the device to the abaxial leaf surface for in situ monitoring. The system detected tomato spotted wilt virus infection four days after inoculation, approximately 6–10 days before visible symptoms appeared. This result supports pre-symptomatic warning in the tested tomato–virus system; the lead time should not be generalized to other pathogens, cultivars, or field conditions [198].

Despite these advances, most surface-mounted wearable VOC sensors detect local concentration changes and therefore do not directly quantify gas-exchange fluxes at the leaf–atmosphere interface. A complementary strategy is to couple individual leaves with lightweight enclosure-based sampling systems. ECOvette, for example, enables long-term, minimally invasive in situ quantification of VOC fluxes from individual leaves under natural field conditions (Figure 13b). By combining inert polymer materials, controlled flow pathways, and high-sensitivity analytical instrumentation, the system enables detection of CO_2_, H_2_O, and trace VOC emissions while minimizing disturbance to leaf physiological activity [199]. Although this configuration differs from conventional surface-mounted wearables, it illustrates the value of directly quantifying plant–atmosphere exchange when volatile flux, rather than local concentration alone, is the physiological variable of interest.

A central advantage emerging from these studies is the possibility of detecting stress-associated chemical changes before clear visual symptoms develop (Table 10). Multichannel sensor arrays combined with pattern recognition can identify changes in volatile profiles directly at or near the leaf surface, allowing individual plants or localized zones to be flagged for further examination. When linked with georeferenced scouting and remote-sensing observations, these alerts can narrow the area requiring confirmation and support earlier, site-specific crop-protection or stress-mitigation measures. The distinction between concentration and flux explains why similar physiological responses may produce different signals under different airflow or enclosure conditions. Humidity and mixed stresses can also alter VOC patterns. Field evaluation should report false alarms and missed detections against independent diagnoses, using plants and dates excluded from model training. VOC sensing is therefore most defensible as a screening layer that directs confirmatory inspection.

**Table 10:** Wearable VOC Sensors for Plant Stress Monitoring.

Target	Technique	Features	Application	Reference
Methanol	PtNPs/poly(ATD)	Wearable, real-time, high catalytic activity	Field stress monitoring	[190]
Multiple VOCs	rGO array	Multi-VOC discrimination	Tomato disease early detection	[195]
α-Pinene	PEVA/MWCNTs	High selectivity	Tree stress monitoring	[200]
Acetone/Hexanal	MWCNTs	Multimodal stress sensing	Multimodal stress sensing	[197]
VOCs	MS detection	Minimal plant disturbance	Ecosystem monitoring	[199]
Ethylene	MXene RF resonator	Wireless; passive sensing	In situ plant ethylene monitoring	[159]

Abbreviations: platinum nanoparticles (PtNPs); poly(2-amino-1,3,4-thiadiazole) [poly(ATD)]; volatile organic compounds (VOCs); reduced graphene oxide (rGO); poly(ethylene-co-vinyl acetate) (PEVA); multi-walled carbon nanotubes (MWCNTs); mass spectrometry (MS); radio-frequency (RF).

### 5.2. Pathogen Detection for Precision Crop Protection

Plant pathogens, including viruses, bacteria, fungi, and oomycetes, continuously threaten crop productivity throughout plant development [201]. During the early stages of infection, these pathogens often induce no visible morphological symptoms while already causing systemic perturbations to host metabolism and defense signaling pathways. Conventional pathogen identification relies on microbial isolation and culture, enzyme-linked immunosorbent assays, or polymerase chain reaction (PCR). Although these techniques provide high analytical accuracy [202], sample transportation, purification, and amplification procedures typically require several hours to several days, causing diagnostic results to lag behind disease spread in the field. Portable molecular and immunological assays can support field diagnosis, but sample preparation and intermittent sampling still constrain continuous surveillance [203,204,205].

The emergence of biosensors has provided a new pathway for translating pathogen detection from laboratory settings to field applications. Their fundamental principle involves integrating biological recognition elements, such as antibodies or nucleic acid probes, with signal transducers. Target recognition is thereby converted into measurable electrical or optical outputs, reducing dependence on extensive labeling and sample-processing procedures. Early miniaturized pathogen-sensing efforts focused on electrode-surface modification strategies [206,207,208]. Antibodies or nucleic acid probes were immobilized onto miniaturizable substrates, including screen-printed electrodes, and target recognition was monitored through interfacial charge variations measured by electrochemical impedance spectroscopy. This approach has been successfully applied to the detection of plant pathogens such as Citrus tristeza virus [209]. These sensors are label-free and operationally simple; however, planar electrode architectures often suffer from insufficient signal-to-noise ratios at low target concentrations, while nonspecific adsorption at electrode surfaces remains difficult to eliminate completely [210,211].

**Figure 14:**
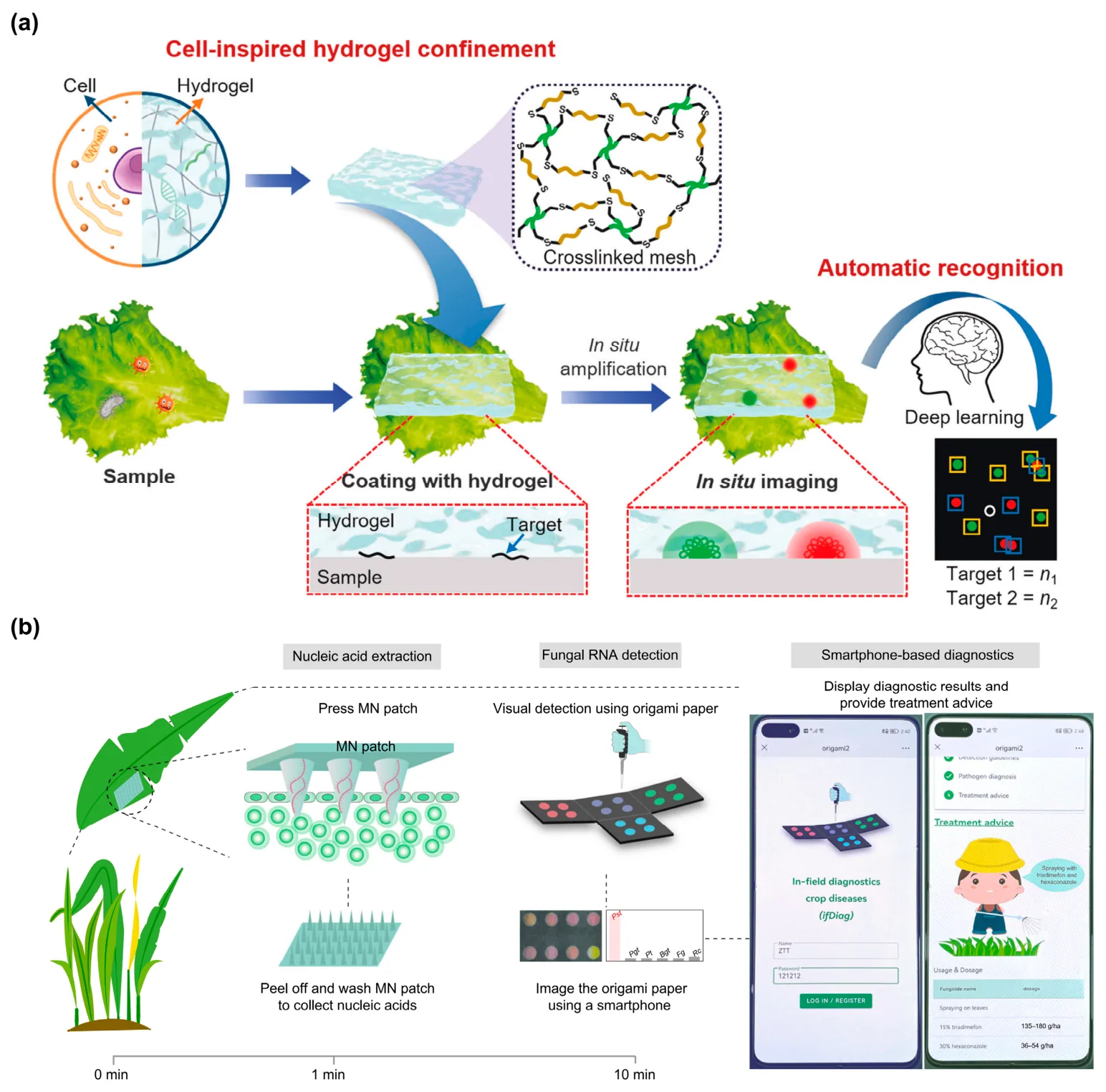
Molecular sensing strategies for plant pathogen detection. (**a**) Hydrogel-based in situ space-confined interfacial amplification (iSCIA) for in situ nucleic acid and pathogen imaging [212]. (**b**) Smartphone-integrated platform for in-field pathogenic RNA diagnosis [219]. Panels (**a**,**b**) are adapted from the respective sources cited above under the terms of the Creative Commons Attribution 4.0 International License (CC BY 4.0).

Advances in functional materials, signal-amplifying interfaces, and transistor architectures have further promoted the miniaturization of pathogen sensors and their integration with plant-compatible platforms. Electrolyte-gated organic field-effect transistor-based immunosensors and molecularly imprinted electrochemical sensors have further improved the sensitivity and miniaturization of pathogen detection by combining signal amplification with selective recognition interfaces. These strategies demonstrate the feasibility of compact pathogen sensors, although their operation generally still requires target transfer to or direct interaction with a predefined sensing interface. To enable direct detection while preserving spatial information, Yang et al. [212] developed a hydrogel-based in situ space-confined interfacial amplification (iSCIA) strategy for nucleic acid and pathogen imaging directly on biological surfaces (Figure 14a). The nanoporous polyethylene glycol hydrogel confines target nucleic acids near their original locations while permitting enzymatic amplification, enabling rapid in situ imaging without conventional extraction or fixation procedures [213,214,215,216,217].

Wearable optical platforms provide another route toward direct pathogen-associated nucleic acid detection on living plants. For example, Kissell et al. [218] developed a reagent-free SERS hydrogel patch capable of detecting tobacco mosaic virus ribonucleic acid (RNA) on tobacco leaves before visible symptoms emerged, demonstrating the potential of plant-attached molecular sensing for pre-symptomatic disease diagnosis. Moving from plant-attached detection toward field-deployable molecular diagnosis, Zhang et al. [219] integrated microneedle-assisted nucleic acid extraction with a paper-based pathogenic RNA assay and smartphone analysis for rapid crop disease diagnosis (Figure 14b). The platform enabled multiplexed detection of wheat fungal pathogens and mutation-resolved analysis of fungicide-resistant isolates, while the smartphone interface translated molecular results into crop-disease management information. This sample-to-result workflow demonstrates how pathogen-specific sensing can be coupled directly with field decision support for precision crop protection.

Unlike stress indicators that infer disease indirectly from physiological or volatile changes, several sensing strategies in Table 11 target pathogen-specific molecular evidence, although the table also includes indirect gas sensing and sample-based assays. Detection of pathogen nucleic acids and other specific molecular signatures before visible symptoms appear can help determine whether anomalies identified by UAV imaging or field scouting are associated with infection, while georeferenced measurements can delineate plants or zones requiring confirmatory sampling and rapid intervention [220]. This combination can support earlier disease mapping, targeted crop-protection measures, and reduced reliance on blanket treatment. Accordingly, the most distinctive role of wearable pathogen sensing is to connect broad-area anomaly detection with pathogen-specific confirmation at the plant level. Field validation must quantify specificity against related pathogens and mixed infections, contamination-related false positives, and false negatives caused by low target abundance or sampling failure. Molecular detection and continuous on-plant monitoring should be evaluated separately [221].

**Table 11:** Wearable Sensors for Plant Disease Detection.

Target	Technique	Key Features	Application	Reference
Citrus tristeza virus	InP/antibody	High sensitivity	Citrus	[209]
Plant viral nucleic acids	AuNPs/DNA probe	Isothermal amplification	Broad-spectrum plant samples	[222]
Plum pox virus	Organic semiconductor/antibody	Label-free; rapid	Stone fruits	[51]
Bean pod mottle virus	Porous polypyrrole	High specificity	Legumes	[223]
Tobacco mosaic virus	Gold nanostar/nucleic acid	Non-destructive detection	Tobacco leaves	[218]
Pepper yellow leaf curl virus	MOS array	Non-contact sensing	Pepper crops	[224]

Abbreviations: indium phosphide (InP); gold nanoparticles (AuNPs); metal oxide semiconductor (MOS).

### 5.3. Pesticide Sensing for Application Assessment and Residue Management

Pesticide residues represent a major risk source for agricultural product safety. During crop growth, plants may be exposed to organic pesticides and metal-based formulations through foliar application and other exposure pathways. Their final residue levels are jointly determined by adsorption on plant surfaces, subsequent penetration, and translocation into internal tissues, which can affect product quality and pose potential risks to human health through dietary exposure [225]. Conventional pesticide detection relies primarily on laboratory-based chromatographic and mass spectrometric techniques [226]. These methods suffer from complex sample pretreatment, long analytical cycles, and an inability to achieve in situ continuous monitoring. In this context, wearable sensors, through flexible attachment or minimally invasive sampling, directly interface with plant surfaces and internal tissues, providing a new technological pathway for rapid and in situ pesticide residue detection [227,228].

Early development of plant wearable pesticide sensors focused on constructing flexible sensing platforms [229]. Zhao et al. [230] fabricated three-dimensional porous graphene electrodes on PI films via laser direct writing and subsequently transferred them onto PDMS substrates to obtain stretchable sensors. The resulting platform enabled wireless detection of methyl parathion on spinach leaves, demonstrating the feasibility of plant-wearable pesticide monitoring. However, petroleum-based substrates such as PI are difficult to degrade, conflicting with the requirements of sustainable agricultural development [231]. To address this issue, Teixeira et al. [232] replaced conventional plastic substrates with cellulose acetate and constructed a carbon-based three-electrode system via solution casting and screen printing. The system enabled sensitive detection of carbendazim and paraquat, demonstrating the potential of biodegradable materials in wearable pesticide sensing for plants. As flexible platforms matured, further improvements in sensing performance became increasingly constrained by the stability of recognition elements [233,234,235].

**Figure 15:**
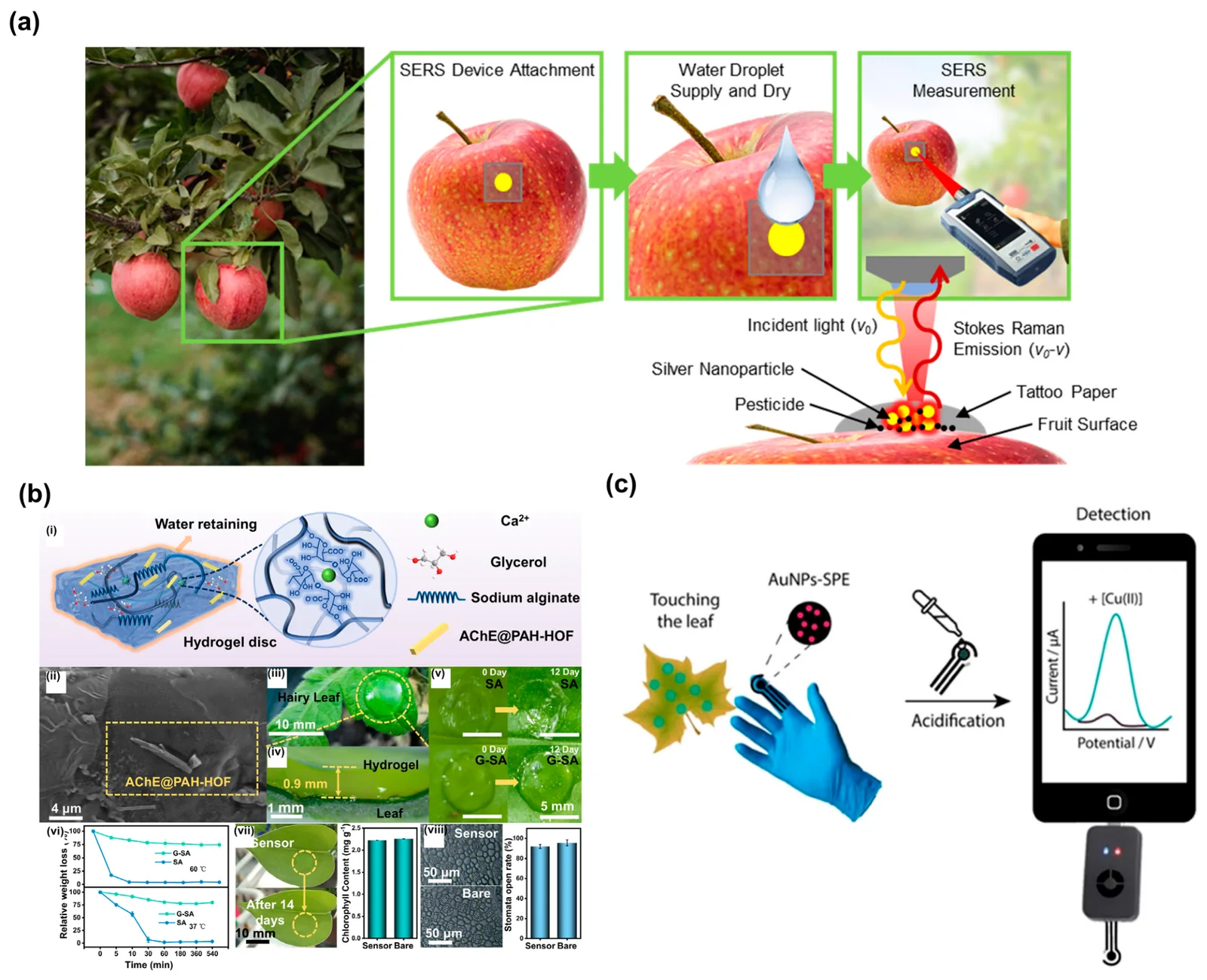
Wearable and surface-adaptable sensors for pesticide monitoring. (**a**) Tattoo paper-based silver nanoparticle substrate for surface-enhanced Raman scattering (SERS) detection of pesticides on fruit surfaces [240]. (**b**) Acetylcholinesterase-based wearable sensor for in situ organophosphate pesticide detection [236]. (**c**) On-glove electrochemical sensor for copper-based pesticide detection on vine leaves [246]. In (**b**), (**i**–**viii**) show hydrogel construction, sensor microstructure, leaf attachment, the contact interface, changes during storage, water retention, chlorophyll measurements, and stomatal morphology/opening, respectively. Here SA denotes sodium alginate and G-SA denotes glycerol-containing sodium alginate; AChE denotes acetylcholinesterase and HOF denotes hydrogen-bonded organic framework. In (**c**), AuNPs-SPE denotes a gold-nanoparticle-modified screen-printed electrode. Panels (**a**–**c**) are adapted from the respective sources cited above under the terms of the Creative Commons Attribution 4.0 International License (CC BY 4.0).

Enzyme-based sensors are widely used because acetylcholinesterase provides a sensitive recognition mechanism for organophosphorus and carbamate pesticides. However, enzymatic proteins are prone to deactivation under field-relevant conditions such as high temperature and ultraviolet irradiation. Li et al. [236] encapsulated acetylcholinesterase within a glycerol–sodium alginate hydrogel using a hydrogen-bonded organic framework (HOF) strategy (Figure 15b). Hydrogen-bond-driven co-assembly preserved the native conformation of the enzyme, and a colorimetric readout enabled in situ detection of chlorpyrifos. The sensor was reported to retain performance over a 13-day evaluation [237]. Recognition-layer stabilization is promising, but stability over the reported test interval does not establish continuous seasonal operation under field weathering [142,238].

While electrochemical platforms continue to improve detection reliability, optical sensing provides complementary advantages through visual outputs and compatibility with smartphone-based readout. Yan et al. [239] embedded upconversion nanoparticles within zeolitic imidazolate frameworks and constructed a polydopamine-containing dual-shell structure integrated into a double-network hydrogel patch. Coupled with smartphone image acquisition and red–green–blue image analysis, the system enabled on-site quantitative detection of 2,4-dichlorophenoxyacetic acid. Mandrekar et al. [240] further developed a flexible tattoo paper-based silver nanoparticle SERS substrate for direct pesticide sensing on fruit surfaces (Figure 15a). Its conformal adhesion to curved apple surfaces enabled rapid Raman identification of thiram residues with minimal sample handling. Extending optical sensing from one-time residue screening toward temporal monitoring, Chen et al. [241] developed a quantum-dot-engineered flexible hydrogel sensor for smartphone-readable thiram detection on multiple crop surfaces. The system further tracked thiram degradation on Chinese cabbage leaves, demonstrating the potential of wearable sensing for evaluating pesticide persistence and pre-harvest residue dynamics [242,243].

Recent studies have also focused on simplifying sample acquisition and on-site analysis so that pesticide measurements can be performed with less external handling. Zhang et al. [244] integrated laser-induced graphene electrodes with passive PDMS microfluidics to construct a plant-wearable platform for autonomous sampling and on-site methyl parathion detection. This design reduced dependence on external liquid handling and demonstrated the value of integrating sample transport with electrochemical readout [245]. A complementary point-of-need strategy was demonstrated by Raucci et al. [246], who integrated a gold-nanoparticle-modified electrochemical strip into a glove for direct detection of copper from copper-based pesticide residues on vine leaves (Figure 15c). The glove-based format simplified surface sampling and enabled rapid residue assessment without conventional sample pretreatment. Beyond improvements in individual sensing platforms, cross-reactive sensor arrays combined with pattern-recognition methods are beginning to enable discrimination and quantification of multiple pesticides, indicating a shift toward multidimensional residue analysis [247].

Together, these developments extend pesticide sensing beyond one-time laboratory residue analysis toward in situ assessment of pesticide deposition, persistence, and degradation on crop surfaces (Table 12). Georeferenced measurements can complement spraying records and weather information by providing plant-level evidence of actual pesticide exposure, while repeated measurements can reveal degradation trajectories after application. Such information is particularly relevant for evaluating treatment outcomes, residue persistence, and pre-harvest management. However, a declining signal may reflect wash-off, diffusion, incomplete sampling, or sensor aging as well as chemical degradation. Surface measurements require validated recovery and sampling-area control, and cannot alone establish residues throughout edible tissues. Their strongest current role is localized post-application screening, with independent analytical confirmation for quantitative residue assessment [248,249,250].

**Table 12:** Wearable Sensors for Pesticide Residue Detection.

Target	Technique	Features	Application	Reference
Methyl parathion	OPH	LIG-based; flexible electronics; wireless operation	Spinach/apple	[230]
Methyl parathion	AChE	rGO/AuNPs; high sensitivity	Leaf surface	[251]
Multiple pesticides	Non-enzymatic	Glove-integrated; multichannel detection	Cabbage/apple	[252]
2,4-D	Fluorescence (enzyme-based)	UCNPs@ZIF@PDA platform	Tomato leaves	[239]
Thiram	Fluorescence (quantum dots)	Dual-color hydrogel; smartphone-readable	Pak choi leaves	[248]
Methyl parathion	Electrochemical	Microfluidic; capillary-driven automatic sampling	On-plant pesticide detection	[244]

Abbreviations: organophosphorus hydrolase (OPH); acetylcholinesterase (AChE); laser-induced graphene (LIG); reduced graphene oxide (rGO); gold nanoparticles (AuNPs); 2,4-dichlorophenoxyacetic acid (2,4-D); upconversion nanoparticles (UCNPs); zeolitic imidazolate framework (ZIF); polydopamine (PDA).

## 6. Challenges and Future Directions for Plant-Wearable-Enabled Precision Agriculture

### 6.1. Reliable Long-Term Operation Under Field Conditions

Field reliability requires stable attachment, calibratable observations, minimal plant disturbance, and sufficient data continuity for the intended management decision. Available analytical work includes toxin assays and optical disease-detection methods [253,254,255,256]. Such evidence supports analytical feasibility but does not establish continuous, integrated field operation. Irrigation, nutrition, and disease-warning systems need time series that resolve onset, persistence, and recovery; sensitivity alone is insufficient.

The plant–sensor interface is intrinsically dynamic. Leaf expansion, stem thickening, fruit enlargement, changes in surface curvature, and the development or redistribution of wax layers continuously modify contact pressure and effective sensing area. Hydrogel patches illustrate one approach to creating adaptable sensing interfaces [257]. Nevertheless, initial adhesion does not guarantee stable measurements throughout a growth stage. Progressive strain can alter resistance, capacitance, mass transfer, or electrode contact independently of the biological variable of interest, producing baseline shifts that may be mistaken for physiological change. Sensor placement and fixation must therefore accommodate organ growth while preserving reproducible coupling between the sensing element and the monitored tissue. Practical service life should be evaluated across vegetative and reproductive stages, since leaf expansion, tissue senescence, and natural shedding can end usable contact before electronic failure. Define inspection and replacement criteria for detachment, constriction, tissue injury, or loss of calibrated response under open-field weather. When moving a device to a replacement leaf or organ, record its position and developmental stage, recalibrate, and flag the transition rather than treating the measurements as an uninterrupted record from the same tissue.

Mechanical conformity must also be considered together with physiological non-interference. A device that remains attached but covers stomata, blocks incident light, restricts tissue expansion, changes the local boundary layer, or damages the cuticle may modify photosynthesis, transpiration, gas exchange, and normal growth, thereby changing the very signals being measured. This concern is particularly important for leaf-mounted temperature, humidity, gas, and optical sensors, and for implanted or microneedle systems that access sap or internal tissues. Field validation should therefore compare monitored and unmonitored organs or plants and report effects on growth, visible injury, stomatal behavior, or other relevant physiological indicators, rather than assuming biocompatibility from material flexibility alone [258,259].

Long-duration measurements are further affected by interacting environmental and biological disturbances. Diurnal temperature and humidity cycles, rainfall, dew, wind, solar radiation, pesticide spraying, dust, and biological contamination can change encapsulation properties, electrode interfaces, and recognition-layer activity. In electrochemical and biochemical sensors, electrode aging, biofouling, reference-potential instability, enzyme deactivation, and adsorption of proteins, polysaccharides, or phenolic compounds can reduce sensitivity and induce drift. Implantation may additionally trigger wound healing, callus formation, or localized lignification, which changes analyte transport near the sensing interface [260]. Because these effects overlap with genuine plant responses, environmental protection alone is insufficient; the system must distinguish sensor degradation from biological variation.

Calibration should cover the intended measurement range and operating temperature and humidity, using standards with stated values, units, uncertainty, and traceability where available. Record calibration levels, replicates, fitted response, residual error, hysteresis, response time, and pre- and postdeployment sensitivity. Temperature and humidity channels require comparison with calibrated reference instruments; chemical sensors require matrix-matched standards or validated recovery tests, blanks, and interferent challenges. Validate physiological interpretation separately, for example against leaf water potential or laboratory tissue-nitrogen measurements. Record sensor placement, sampling interval, equilibration time, and recalibration frequency. Establish environmental compensation using controlled tests and retain raw readings alongside corrected values. Reference-channel excursions or sensitivity loss beyond a prespecified decision-error budget should trigger inspection, recalibration, or replacement. Do not remove unexplained biological changes as drift. Fouling controls and cleaning require validation of analyte transport, response time, and plant injury; affected intervals should be flagged. For electrochemical systems, assess reference-electrode potential separately against an independently checked reference in a controlled electrolyte before deployment and at maintenance intervals justified by observed stability. Where this check requires device removal, document the interruption and recalibration. Channels sharing one reference cannot independently identify its drift; a redundant channel is informative only if its reference stability is separately established. Apply offset corrections only when supported by reference checks, and otherwise flag affected intervals and increase uncertainty rather than inferring drift from the plant signal alone.

Self-powered photoelectrochemical platforms explore light-driven analytical signal generation [261,262]. This does not establish energy autonomy for an entire wearable network. Battery or harvesting budgets must include conditioning, computation, storage, and wireless transmission under variable weather. Duty cycling and event-triggered acquisition should be evaluated against the temporal resolution needed to detect the target response.

Optical monitoring approaches also differ in their acquisition and interrogation requirements [263]. Related analytical work includes a self-powered photoelectrochemical assay [264]. This does not demonstrate a self-powered communication node. Network validation should separately test canopy-related link loss, local buffering, retransmission, and clock synchronization. Select communication protocols using field size, latency, energy use, and maintenance requirements; provide missing-data flags and a fallback when observations are unavailable.

Operando characterization can clarify changes at sensing interfaces [265]. Field validation requires additional system-level evidence: operating duration, baseline and sensitivity drift, valid-data fraction, calibration interval, node failures, maintenance, cross-plant repeatability, and effects on growth. Report these across relevant phenological stages and weather conditions. Bench cycling or interface characterization cannot substitute for seasonal deployment tests.

### 6.2. Precise Multiparameter Sensing for Crop Physiological-State Assessment

Agricultural sensing encompasses molecular pathogen assays and machine-vision weed detection [266,267]. Multiparameter sensing should not aim to maximize the number of channels, but to reduce physiological ambiguity by combining complementary signals related to a predefined crop-state variable. Redundant channels that respond to the same interference may increase system complexity without improving interpretation, whereas appropriately selected physical, chemical, and biochemical measurements can distinguish transient environmental effects from persistent physiological change. Multiparameter platform design should consequently shift from device-driven integration toward state-oriented sensing [268,269].

The target state should have a measurable reference, unit or diagnostic definition, spatial support, and prediction horizon. For irrigation, estimate water potential or stomatal conductance and its recovery trajectory; for nutrition, estimate a defined tissue nutrient measure; for disease, estimate the probability of a specified infection against diagnostic results. Broad labels such as “healthy” or “stressed” obscure different causes and management needs. Phenotyping and stress-classification studies illustrate the diversity of possible endpoints [270,271]. Related analytical and fusion methods offer useful tools, but their outputs require physiological validation before management use [272,273,274].

Sensing limits and management thresholds serve different purposes. A detection limit characterizes an analytical method; it is not an irrigation trigger or a disease-action threshold. Define decision thresholds in the target variable’s units, or as a calibrated probability, using crop- and growth-stage-specific reference measurements and the consequences of missed or unnecessary interventions. Prespecify the persistence window, uncertainty margin, minimum valid-data coverage, and recovery condition. For example, a brief midday leaf-temperature rise should not trigger irrigation without environmental compensation and corroborating water-status evidence. This review proposes reporting and validation requirements rather than universal numerical settings for heterogeneous crops and devices.

Each stream should retain its unit, timestamp, response time, sensor position, calibration status, and missing-data flags. Optical and spectroscopic studies illustrate how crop traits depend on measurement context [275,276]. Analytical platforms also differ in readout chemistry and sample matrix [277,278,279].

Hyperspectral nutrient-estimation studies provide spatially distributed trait estimates that can complement wearable measurements [280,281]. For asynchronous fusion, define windows from the target process: for example, summarize rapid temperature observations over a prespecified short irrigation-assessment window, while retaining UAV observations at acquisition time. Store the age and coverage of each estimate. Apply sensor response-delay corrections established by calibration, and estimate physiological lags only within training data. Do not create apparent high-frequency information by replicating sparse measurements across timestamps.

Related work spans optical and electrochemical assays, seed-vigor fusion, and hyperspectral trait estimation [282,283,284]. For the proposed wearable workflow, retain native timestamps and summarize only valid observations within each window. Require minimum coverage, distinguish communication loss from sensor failure, and mark interpolated values explicitly. Short gaps may be estimated with validated uncertainty; long gaps or stress transitions should remain missing. These rules separate temporal alignment from the choice of fusion model.

Here, early fusion combines calibrated observations on a common time support before feature extraction; intermediate fusion combines separately derived features; and late fusion combines modality-specific state estimates or probabilities. Early fusion is appropriate for synchronized channels measuring coupled processes, but is vulnerable to missing values and correlated interference. Intermediate fusion may be appropriate when thermal, strain, and biochemical streams require different preprocessing and cross-modal features contribute useful information. Late fusion is useful for asynchronous or intermittently available sources, provided their outputs estimate the same target at a compatible scale and horizon. Weights should reflect validated predictive uncertainty and cross-modal dependence. Late fusion cannot correct an unresolved leaf-to-field mismatch, and its tolerance of sensor dropout must be tested explicitly.

Fusion should be benchmarked against the best single modality and an environmental-data baseline, using ablation to test the additional value of each channel. Split training and testing by plant, site, or season rather than randomly separating adjacent timestamps. Fit normalization, lag selection, and feature selection within training folds to prevent leakage. For continuous states, report bias, error, and interval coverage; for disease alerts, report sensitivity, specificity, and precision at the intended prevalence. Test stage transfer, missing channels, and sensor drift separately. Multimodal analytical work offers transferable concepts [285]. These concepts still require validation on living plants under the intended deployment conditions; analytical fusion performance alone does not establish agronomic benefit.

For high-dimensional spectral inputs, report wavelength range and resolution, illumination and viewing geometry, integration time, calibration frequency, and region-of-interest selection. Apply dark and reference-panel correction to reflectance images, mask soil and background pixels, and document removal of saturated or low-signal bands. If smoothing, derivatives, scatter correction, or normalization are used, specify their order and parameters; excessive smoothing can erase diagnostic features. Compare full-spectrum models with selected bands or extracted components. Fit all data-dependent preprocessing, feature selection, and model tuning within training folds. Report algorithm and software versions, hyperparameter search space, selected settings, random seed, fold count, and grouping unit. Use nested grouped cross-validation for tuning and reserve independent plants, sites, or seasons for final testing; pixels and repeated spectra from one plant must remain in the same partition. Report prediction error in the reference measurement’s units and test transfer across illumination, humidity, and soil conditions.

For precision agriculture, the principal contribution of multiparameter wearable sensing is therefore to generate physiologically interpretable plant-centered state variables that cannot be obtained directly from soil, meteorological, or canopy observations. These variables can describe the magnitude, progression, and recovery of crop responses and provide a physiological layer between raw sensor signals and field management. Their practical value, however, ultimately depends on whether observations from selected plants can represent spatial variation across management zones and be integrated with soil, weather, and remote-sensing information, which is addressed in the following section [286].

### 6.3. Scaling from Individual Plants to Field-Level Management

Physiologically interpretable crop-state estimates remain measurements of particular organs or individual plants. Their contribution to precision agriculture depends on whether these observations can represent the spatial variability relevant to field management. This transition cannot be achieved by directly extrapolating a response recorded from one leaf or plant to an entire field. Variability in genotype, developmental stage, canopy position, root-zone conditions, topography, microclimate, and previous management can cause plants within the same field to exhibit different physiological responses. Scaling plant wearable sensing therefore requires an explicit sampling framework that links monitored plants to the spatial units at which agricultural decisions are made [287].

Representativeness must first be considered within the plant itself. Measurements obtained from a single leaf may vary with leaf age, orientation, illumination, canopy position, and local airflow, while stem- and fruit-mounted sensors characterize different physiological processes and temporal responses. Sensor location should consequently be selected according to the target crop state, and the monitored organ, canopy position, developmental stage, and installation protocol should be recorded [288,289,290,291]. Where substantial within-plant variability is expected, measurements from multiple organs or repeated placement on comparable organs may be needed before a plant-level state can be assigned. This distinction is important because measurement precision at one attachment site does not necessarily imply accurate characterization of the whole plant.

Partition the field into management zones using soil properties, elevation, irrigation layout, cultivar, and historical or current canopy observations. Within each zone, select replicate plants using random or spatially balanced sampling, rather than choosing only convenient or visually average plants. Keep targeted high-risk nodes separate from the probability sample: they can support early warning but should not be averaged into zone estimates without an explicit sampling model. Record installation criteria and replacement rules so that loss or relocation of a node does not silently change the sampled population.

We propose physiological sentinel nodes as a deployment concept requiring validation. Their purpose is to supply continuous physiological observations from sampled plants and test whether canopy or environmental anomalies correspond to persistent plant responses. They are not presumed to represent a field by virtue of carrying a sensor. Representativeness must be established for the target variable, organ, developmental stage, and management zone, and reassessed as the crop develops. The term describes a proposed role within a monitoring network, not a mature field-scale solution.

Plant wearable sensors should therefore be integrated with, rather than positioned as replacements for, existing precision-agriculture information sources. Soil sensors characterize root-zone water, nutrient, salinity, and temperature conditions; meteorological observations describe external atmospheric forcing; and remote sensing identifies spatial variation in canopy structure, temperature, reflectance, and growth. Wearable sensors add high-frequency observations of the plant response itself, while crop models provide a framework for estimating unobserved states and simulating future conditions. These sources answer different questions and operate at different spatial and temporal resolutions. Their combination can distinguish resource availability from crop response and relate detailed physiological observations from selected plants to broader spatial patterns [292,293,294].

One practical pathway is to use wearable observations to calibrate or interpret canopy-scale indicators within defined management zones. Remote sensing may identify areas with contrasting canopy temperature, spectral response, or growth, but such differences do not uniquely reveal their physiological cause. Continuous measurements from sentinel plants can help determine whether these spatial patterns coincide with persistent changes in plant water relations, nutrient-associated responses, growth, or stress progression. Conversely, remote-sensing and soil information can indicate whether a response observed at one wearable node is localized or shared by a larger area. This reciprocal interpretation is more defensible than assuming that either an individual plant or a canopy image independently represents field condition [295,296].

Cross-scale integration requires standardized data structures. Each measurement should retain its timestamp, unit, calibration method, sensor type, monitored organ, attachment position, plant identifier, cultivar, developmental stage, and spatial coordinates. Quality-control flags, missing-data records, replacement history, and uncertainty estimates should accompany the observations. Without such metadata, signals collected from different plants, instruments, or dates cannot be reliably compared or assimilated into field-level models. Interoperability with farm management information systems and decision-support platforms is equally important, because physiologically meaningful data have limited operational value when they cannot be linked to management-zone boundaries, irrigation records, fertilizer applications, crop-protection treatments, or subsequent crop responses.

Node allocation should begin with a pilot study that partitions variability among management zones, plants, and organs within plants. Repeated timestamps are not independent biological replicates. Determine the number of sentinel plants from between-plant variability, the precision required for the management decision, spatial correlation, and expected node failure. Where within-plant variability is substantial, evaluate additional organ positions alongside additional plants; concentrating sensors on fewer plants can reduce field coverage. Test network estimates against independent validation plants using prespecified prediction-error limits and, where appropriate, equivalence margins linked to the management threshold. A nonsignificant difference alone does not establish representativeness. Add or reallocate nodes when validation criteria fail. Disease surveillance requires a separate design accounting for prevalence and acceptable missed-detection risk.

Economic and operational feasibility must be evaluated at the network level. Relevant costs include not only sensor fabrication, but also installation time, the number of nodes per hectare, calibration, communication, data storage, maintenance, replacement after failure, and removal or reuse after deployment. Highly informative measurements may still be impractical if sensors require frequent manual recalibration or replacement. Conversely, a limited number of reusable sentinel nodes may be viable when they materially improve the timing or spatial targeting of field inspection and management. Evaluation should therefore compare the additional information obtained with its total deployment cost and with the agronomic consequences of delayed, missed, or unnecessary interventions.

A defensible scaling workflow therefore combines stratified deployment, organ-level calibration, and independent validation of zone estimates. Hierarchical models can retain organ- and plant-level variability instead of treating every timestamp as a separate field observation. Zone estimates should carry sampling uncertainty and, where needed, design weights. Remote-sensing patterns can help identify unsampled conditions, but correlation with a canopy index does not prove field representativeness. Scaling remains a testable inference problem even when individual devices operate reliably.

### 6.4. Data-Assimilated Prediction and Adaptive Management in Precision Agriculture

The system boundary for the proposed crop digital twin includes sampled organs and plants, their root-zone soil and local atmosphere, and the management zone receiving an intervention. Define cultivar, growth stage, rooting depth, spatial resolution, forecast horizon, update interval, and irrigation capacity before calibration. Weather and irrigation are inputs; water status, nutrient status, and growth are distinct states or outputs requiring separate validation. Soil texture, moisture, salinity, and temperature should enter calibration or stratification where relevant, rather than being absorbed into an unexplained sensor offset. Leaf temperature requires radiation, airflow, leaf position, and growth-stage context, with a calibrated energy-balance relationship or justified aggregation of repeated leaf observations before canopy-level assimilation. A local measurement should support local diagnosis when this mapping is unvalidated.

A digital twin adds a repeated forecast–observation–update–decision cycle to this model framework. Existing agricultural modeling and digital-twin studies provide context [297,298,299,300]. For a proposed wearable-enabled implementation, an ensemble Kalman filter is a practical candidate when ensemble spread adequately describes uncertainty and the update is approximately Gaussian. Propagate weather and parameter uncertainty through the forecast, compare predicted and measured observations using the calibrated relationship, and update only physiologically linked states. The observation-error covariance should include calibration, residual drift, and spatial representativeness, with dependence among channels retained. Assimilate asynchronous measurements when they arrive; repeated high-frequency samples must not be counted as independent evidence. Persistent biased innovations should trigger diagnosis of the sensor or observation model rather than progressively stronger corrections.

Irrigation and physiological-response studies provide management endpoints against which plant-based feedback can be tested [301,302,303]. As a test case, combine leaf temperature and strain with soil water and weather, estimate a validated water-status variable, and simulate candidate irrigation times and amounts. Apply limits on available water and excessive wetting, then compare predicted recovery with post-irrigation measurements. Nutrient concentrations need tissue and uptake context before fertilizer adjustment, while nonspecific VOC or temperature alerts should trigger diagnostic scouting before crop-protection treatment. These are proposed workflows, not demonstrated outcomes of every cited sensor.

Precision irrigation and nutrient-estimation studies address different intervention and observation problems [304,305]. Evaluate wearable-assisted management against both a model without wearable updates and the current management practice. Use independent sites or seasons, report forecast error and uncertainty coverage, and measure decision outcomes such as water use, yield, or unnecessary treatments. Low-confidence, conflicting, or out-of-domain observations should prompt additional sampling or a return to the established management rule. Model updating alone is not evidence that autonomous intervention improves crop performance.

These two sources contribute different parts of the proposed workflow: the sprinkler-irrigation review informs water-delivery constraints and scheduling, whereas the lettuce terahertz/near-infrared study informs indirect nutrient observation. Neither establishes a validated wearable-driven crop digital twin. Consequently, the proposed architecture separates state estimation from intervention: spectral estimates require independent tissue-reference calibration and transfer testing, while irrigation decisions must respect delivery capacity and soil-water conditions. A good nutrient-prediction model does not by itself justify a fertilizer dose, and a successful irrigation controller does not validate leaf-scale physiological inference.

Digital-twin applications span machinery and greenhouse systems [306,307]. Their existence should not be taken as validation of a physiological digital twin driven by plant wearables. Such a system remains a research pathway until observation mapping, representative sampling, and agronomic benefit are demonstrated together.

Machinery twins model component loads, wear, faults, and maintenance actions; their principal outcomes concern equipment reliability and downtime. Environmental and crop-production twins instead represent microclimate, soil–plant exchanges, growth, resource use, or yield. Greenhouse-level climate control is relevant architectural context, but does not automatically validate organ-level physiological prediction. The present review focuses on the latter agricultural states and the measurements linking them to management decisions. Machinery-maintenance studies contribute general ideas about synchronization and fault handling, not evidence for crop-response models or agronomic thresholds.

## 7. Conclusions

Readiness is uneven across targets and deployment conditions. Thermal, humidity, and strain wearables offer the clearest near-term route to continuous plant-response monitoring, but their physiological ambiguity still requires environmental context and independent calibration. Ion, hormone, and metabolite sensors provide more specific chemical information, while reference stability, extraction, and tissue responses constrain extended deployment. VOC and acoustic platforms are promising screening tools; pathogen-specific assays can strengthen confirmation but do not necessarily provide continuous monitoring. These are qualitative assessments of the reviewed evidence, not standardized technology-readiness scores.

The next step is a staged test of physiological sentinel nodes: define one management target, calibrate organ-level measurements, estimate within- and between-plant variability, validate replicated zone sampling, and only then test model-assisted decisions. Field trials should compare environmental monitoring alone with the addition of wearable feedback across growth stages and seasons. Success requires lower decision error or improved water, nutrient, or crop-protection outcomes at acceptable maintenance cost and without measurable plant harm. More sensing channels and more complex models are justified only when they add independently validated information.

## Data Availability

No new data were created or analyzed in this study. Data sharing is not applicable to this article.
